# Beyond Tea: Kombucha SCOBY as a Starter Culture Across Food Matrices

**DOI:** 10.3390/molecules31183309

**Published:** 2026-09-18

**Authors:** Muhammad Salman Farid, Monika Sienkiewicz, Łukasz Łopusiewicz

**Affiliations:** 1 Department of Pharmaceutical Biology, Institute of Pharmacy, University of Greifswald, Friedrich-Ludwig-Jahn-Str. 17, 17489 Greifswald, Germany; muhammadsalman.farid@uni-greifswald.de; 2 Department of Pharmaceutical Microbiology and Microbiological Diagnostics, Medical University of Lodz, 90-151 Lodz, Poland; 3 School of Medical and Health Sciences, VIZJA University, Okopowa 59, 01-043 Warszawa, Poland

**Keywords:** SCOBY, kombucha, non-tea food fermentation, acetic acid bacteria, yeast, defined starter cultures

## Abstract

The kombucha symbiotic culture of bacteria and yeast (SCOBY) is a multispecies microbial consortium distributed between cell-containing fermentation liquid and a cellulose-rich pellicle. Here, “kombucha SCOBY” denotes the viable consortium delivered through either compartment or both compartments. Dominated by fermentative yeasts and acetic acid bacteria, the consortium produces ethanol and organic acids and biotransforms phenolic compounds, enabling fermentation beyond conventional sweetened teas. This review critically evaluates studies in which viable consortia directly fermented food matrices. In dairy systems, kombucha-derived inocula acidify milk and induce casein gelation, although more slowly than yogurt starters, and kombucha broth has also supported fresh cheese production. In soy-based matrices, fermentation converts isoflavone glucosides to aglycones, and fermented soy whey can coagulate tofu. Applications have also been reported in fruit and vegetable juices, agro-industrial by-products, botanical infusions and breadmaking. However, microbial composition and performance vary with the inoculum source, propagation history, and storage. Strain-defined preserved starters remain unvalidated in non-tea matrices, and potential hazards include mycotoxin formation and post-production ethanol accumulation. Therefore, standardized, comprehensively characterized, and matrix-specific starters are required for safe and reproducible translation across food matrices.

## 1. Introduction

**Figure 1:**
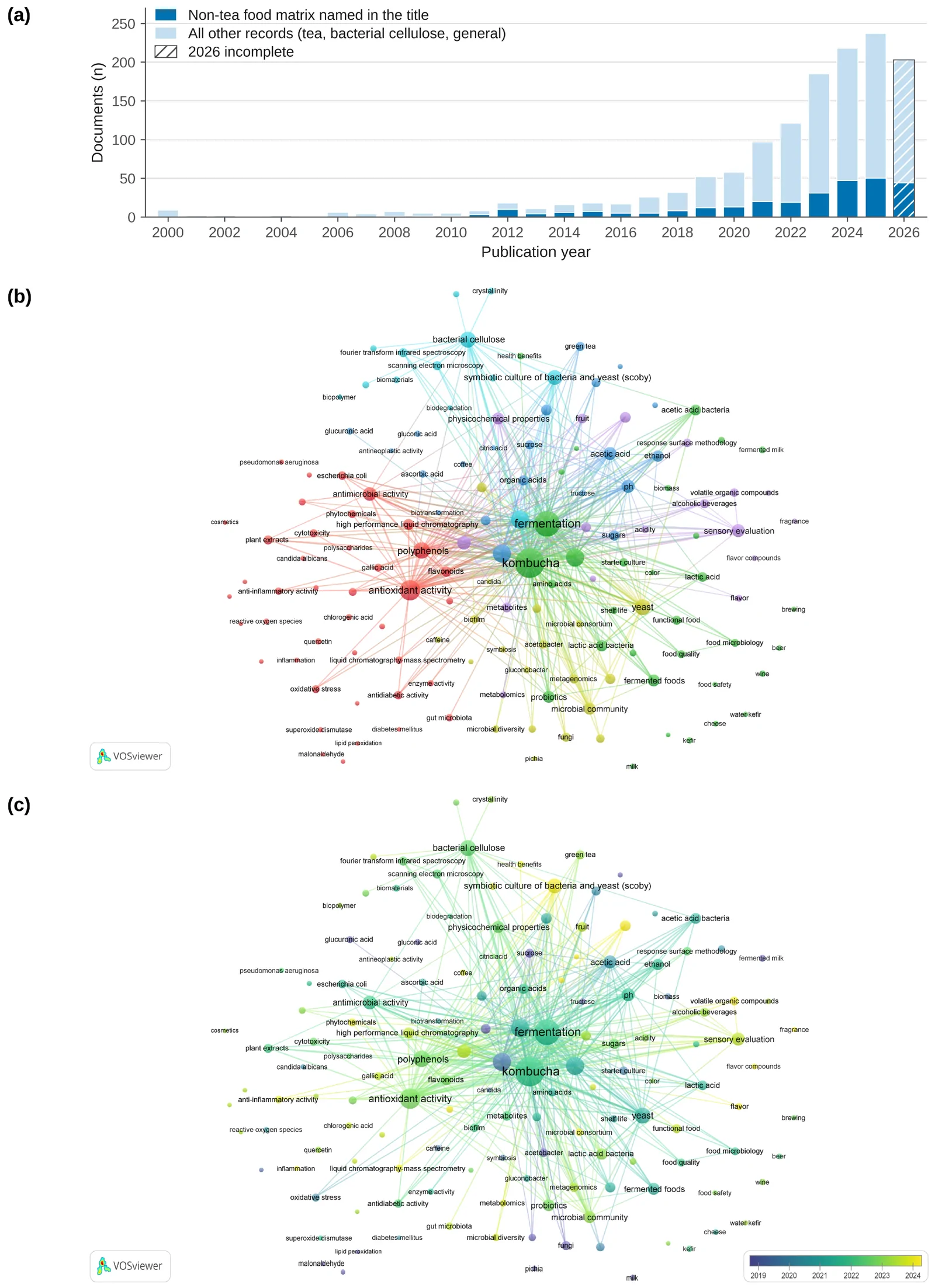
Bibliometric profile of kombucha fermentation research indexed in Scopus (*n* = 1370; retrieved 4 September 2026). (**a**) Annual publication output, 2000–2026; eight documents published before 2000 are not shown, and the 2026 bar is hatched because indexing was complete only to the retrieval date. The dark segment of each bar denotes documents whose title names a non-tea food matrix, and the light segment denotes all other records. (**b**) Keyword co-occurrence network: node size is proportional to occurrence frequency, edge thickness to co-occurrence strength, and color to cluster membership. (**c**) The same map in overlay visualization, where the color denotes the average publication year of the documents in which each keyword appears. Maps were generated using VOSviewer 1.6.20.

Kombucha is a traditional fermented tea beverage produced by a multispecies microbial consortium, conventionally termed a symbiotic culture of bacteria and yeasts (SCOBY), or “tea fungus” [1]. The consortium is distributed between two interconnected compartments: the cell-containing fermentation liquid and the cellulose-rich pellicle formed at the air–liquid interface. In this review, “kombucha SCOBY” is used operationally as an umbrella term for the viable microbial consortium delivered through cell-containing fermentation liquid, pellicle-associated biomass, or both; it does not refer to purified bacterial cellulose or to cell-free kombucha metabolites. Traditional propagation uses a cellulose-rich pellicle and cell-containing fermentation liquid from a previous fermentation as the starter for a new batch [2]. Global commercial interest in kombucha has surged in recent years, and the recognition of the technological and metabolic versatility of the consortium beyond tea has stimulated research into alternative food matrices (Figure 1a).

SCOBY is a microbial consortium whose metabolic output is determined by its coexisting constituent species. Yeasts hydrolyze sucrose and ferment sugars to ethanol, and acetic acid bacteria (AAB), including *Komagataeibacter* species, oxidize ethanol to acetic acid and glucose to gluconic and glucuronic acids [1,3]. During fermentation, organic acid accumulation lowers the pH, and the final value depends on the culture, substrate, inoculum, and process conditions. The resulting acidic environment suppresses many competing microorganisms while selecting acid-tolerant taxa, such as *Zygosaccharomyces bailii* and *Brettanomyces bruxellensis* [4]. This consortium also secretes enzymes that biotransform substrate polyphenols [3]. Antimicrobial activity is largely attributable to organic acid accumulation and low pH, although residual activity after neutralization has been reported and may reflect other metabolites [2,5].

These attributes make SCOBY conceptually attractive as a multi-species starter culture for diverse food matrices. Unlike single-strain or defined binary starters, a wild symbiotic consortium provides concurrent acidification, ethanol production, exopolysaccharide synthesis, and enzymatic biotransformation, potentially combining several transformations in a single inoculation. The intrinsic antimicrobial potential of the consortium against Gram-positive and Gram-negative pathogens, as well as fungal pathogens such as clinical *Candida* isolates [5], further suggests its utility as a candidate bioprotective starter. However, the wild consortium is unstable in storage. In one stored herbal kombucha, acetic acid bacteria were lost at room temperature and the yeast composition shifted [6]. Rationally assembled synthetic microbial communities can reproduce the selected metabolic and sensory outputs of traditional kombucha under controlled conditions [7,8]. Recent research has extended this approach toward function-directed strain selection, controlled consortium assembly, optimization of inoculation ratios, and starter preservation. However, these advances remain predominantly tea-based and laboratory-scale, and evidence of reproducible performance in non-tea food matrices is lacking.

Several reviews have examined kombucha produced from alternative raw materials [9,10,11,12], and a recent review synthesized how fruit, vegetable, herbal, algal, and by-product substrates shape the bioactive properties of kombucha beverages [13]. SCOBY-derived polysaccharides and selected food uses of the culture have also been reviewed [14]. These reviews are largely beverage- and bioactivity-oriented and treat substrates as units of analysis. In contrast, the viable consortium itself has received less focused treatment as a starter culture for non-tea foods, including dairy, soy, and cereal matrices, particularly with respect to inoculum format, matrix-specific technological performance, compound-level chemical characterization, reproducibility, preservation, and safety. Therefore, this review critically evaluates peer-reviewed studies in which viable kombucha-derived inocula directly fermented food matrices. Studies involving purified bacterial cellulose, cell-free products, or kombucha added only after fermentation are considered adjacent applications rather than core evidence. Section 2 establishes the microbial, biochemical, and structural foundations of this consortium, and Section 3 examines the analytical basis on which its chemistry is characterized, together with the molecules reported across tea and non-tea fermentations. Section 4, Section 5, Section 6, Section 7 and Section 8 discuss substrate-specific applications: dairy fermentation (Section 4), plant-based milks and soy-derived foods (Section 5), fruits, vegetables, juices, and agro-industrial by-products (Section 6), bakery and cereal-based foods (Section 7), and coffee, cocoa, and botanical infusions (Section 8). Section 9 addresses reproducibility and process control through the use of defined starter cultures and preservation. Section 10 evaluates safety hazards, regulatory frameworks, and evidence of adverse events.

### 1.1. Literature Search Strategy and Selection Criteria

Literature was identified in Scopus, Web of Science Core Collection, PubMed, and ScienceDirect, supplemented by Google Scholar and manual screening of the reference lists of all included studies and earlier kombucha reviews. Three blocks were combined with AND: (“kombucha” OR “tea fungus” OR “SCOBY” OR “symbiotic culture of bacteria and yeast*” OR “*Medusomyces gisevii*”) AND (ferment* OR “starter culture” OR inocul* OR “co-culture” OR pellicle) AND (milk OR yogurt OR cheese OR whey OR dairy OR soymilk OR tofu OR “plant-based milk” OR juice OR fruit OR vegetable OR pomace OR “by-product” OR “agro-industrial” OR bread OR sourdough OR cereal OR buckwheat OR coffee OR cocoa OR herbal OR botanical OR infusion OR “non-tea”), searched in the title, abstract, and keyword fields. No publication year limit was applied (corpus 1995–2026; 68% of cited sources from 2020 onward), and only English full texts were included.

Eligible studies were peer-reviewed primary research in which a viable kombucha-derived inoculum, fermentation liquid, pellicle biomass, back-slopped broth, or a consortium reconstructed from kombucha isolates fermented a non-tea food matrix. Studies using non-viable material (purified bacterial cellulose, cell-free supernatants, extracts, pasteurized or lyophilized kombucha added after fermentation), kombucha used as an ingredient or coating rather than as the fermenting agent, non-food cellulose applications, and non-peer-reviewed items were excluded. Reviews and standards served only for contextual framing, and every quantitative value was verified against the primary articles. As a narrative review, no risk-of-bias instruments or pooling were applied.

### 1.2. Bibliometric Overview

The broad Scopus corpus (*n* = 1370; English articles, reviews, and conference papers, 1995–2026) was analyzed for annual output (Figure 1a) and mapped in VOSviewer 1.6.20 (all keywords, full counting, minimum 10 occurrences, synonyms merged by thesaurus; 146 keywords, 6 clusters), presented as the cluster visualization in Figure 1b and the average-publication-year overlay in Figure 1c. Annual output increased from 18 documents in 2015 to 237 in 2025, and 82% of the corpus (*n* = 1119) was published from 2020 onward, indicating a rapidly expanding and recent field. Growth in non-tea work has been proportional rather than disproportionate: documents naming a non-tea food matrix in the title increased from 7 in 2015 to 50 in 2025, yet their share of annual output remained close to one-fifth throughout (288 of 1370 records, 21.0%).

## 2. The SCOBY as a Microbial and Biochemical System

Kombucha SCOBY harbors a phylogenetically diverse consortium in which acetic acid bacteria (AAB) and osmotolerant yeasts occupy complementary metabolic niches. High-throughput amplicon surveys consistently place *Komagataeibacter* (formerly *Gluconacetobacter*) as the dominant bacterial population in both liquid medium and biofilms, with additional contributions from lactic acid bacteria, such as *Lactobacillus* and *Lactococcus* [1]. The yeast compartment is variable: culture-dependent and sequence-based surveys identify *Zygosaccharomyces bailii*, *Brettanomyces bruxellensis*, *Schizosaccharomyces pombe*, and *Torulaspora delbrueckii* among the predominant species, with *Z. bailii* typically dominating due to its exceptional tolerance to high-sugar and acetic acid environments, reaching up to 84.1% relative abundance in alternative substrates such as coconut water [1,4]. Community succession follows a pattern observed in some tea fermentations: osmotolerant species initiate the process and are progressively outcompeted by acid-tolerant taxa [4].

**Figure 2:**
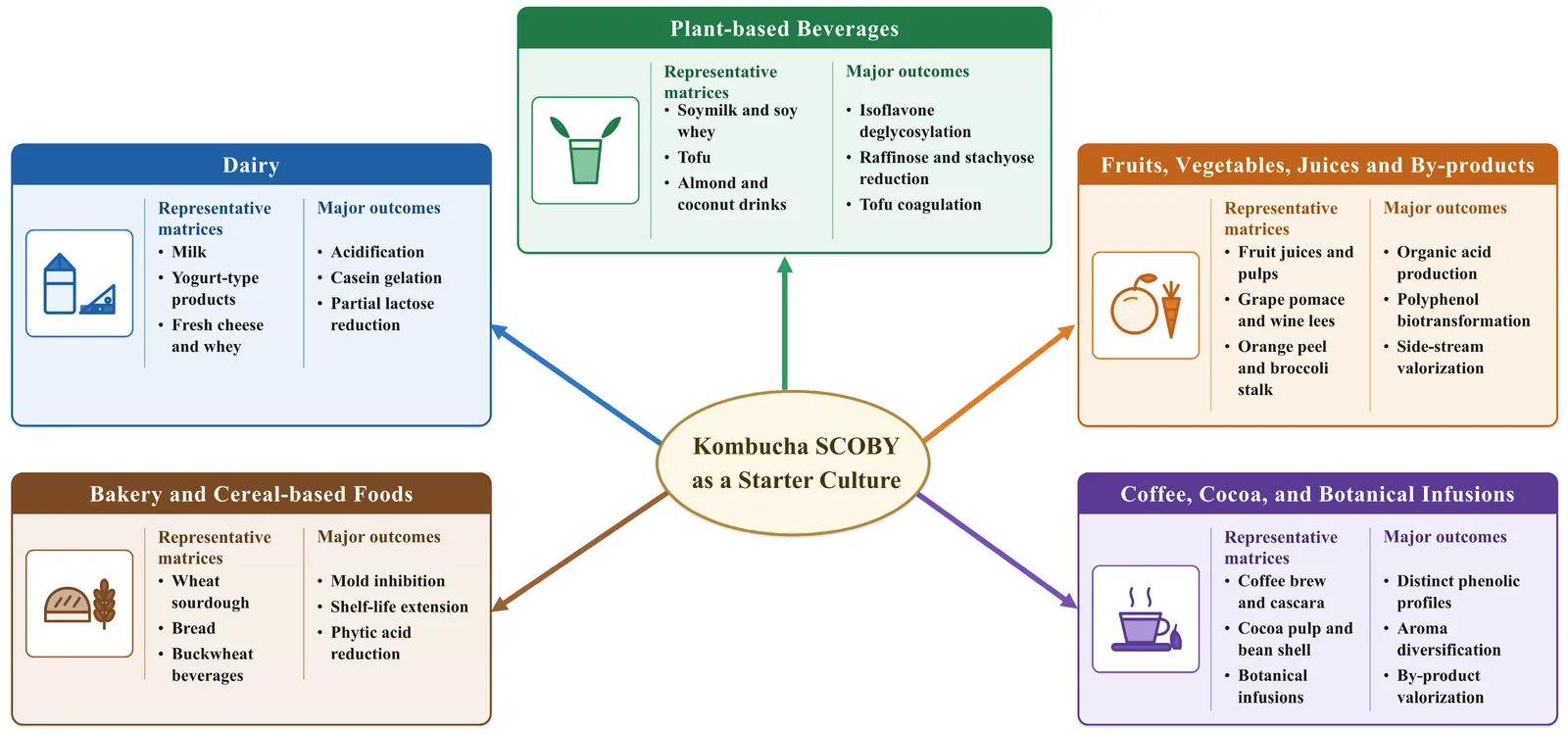
Non-tea food applications of the kombucha SCOBY as a starter culture across food matrices.

This trophic architecture drives sequential biochemistry. Yeast invertase hydrolyzes sucrose into glucose and fructose; glycolytic conversion yields ethanol, which is oxidized to acetic acid by AAB, a reaction that simultaneously stimulates further yeast ethanol production, establishing a positive feedback loop [1,2]. Glucose is additionally channeled to gluconic and glucuronic acids via bacterial oxidation at C-1 and C-6, respectively [3]. Concurrently, catechins (EGCG and ECG) are degraded during fermentation; however, the total phenolic content increases (up to 27%) as complex polyphenols are biotransformed into simpler derivatives [2,3]. Battikh et al. [5] found that kombucha analogues inhibited seven bacterial pathogens, with *Lippia citriodora* ferment being especially active against *Listeria monocytogenes*, and suppressed clinical *Candida* isolates. Neutralization abolished most of the antibacterial potency, confirming organic-acid pH depression as the primary mechanism. This positions the consortium as a potential biopreservation agent beyond tea. The consortium is spatially distributed between the cellulose-rich pellicle and liquid phases, with microbial abundance and composition potentially differing between these compartments. The non-tea food matrices in which the consortium has been applied as a direct starter, along with their representative substrates and principal outcomes, are summarized in Figure 2.

## 3. Chemical Analysis and Molecular Composition of Kombucha SCOBY Fermentations

Molecular characterization of kombucha SCOBY fermentation relies on a limited set of analytical platforms, the resolution of which differs by orders of magnitude; therefore, platform selection determines the scope of interpretation. Ion-exclusion and reversed-phase high-performance liquid chromatography (HPLC), coupled with refractive index, UV, or diode-array detection, are standard methods for quantifying organic acids, sugars, and ethanol, although columns and mobile phases vary across studies [3,8,15,16,17,18]. Enzymatic assays are also used to quantify these analytes in dairy and by-product matrices and for regulatory ethanol determination, for which an AOAC-validated method is available [19,20,21]. Volatile compounds are resolved by gas chromatography–mass spectrometry following headspace or stir-bar sorptive extraction [22,23,24]. Identification beyond predefined targets requires mass spectrometry or NMR. Applications include UPLC–MS^E^ for untargeted phenolic profiling of black tea kombucha [25], HPLC–PDA–ESI/MS^n^ combined with UHPLC–ESI–QqQ–MS/MS for glucosinolates and their isothiocyanate hydrolysis products [26], and ^1^H NMR for metabolite fingerprinting of kombucha sourdough starters [27]. Minerals are determined using flame atomic absorption spectrometry [28]. However, the measurements most frequently reported in this field do not resolve individual compounds. Folin–Ciocalteu estimates of total phenolic content, expressed as gallic acid equivalents, and radical-scavenging or reducing power assays (DPPH, ABTS, FRAP, CUPRAC, and PCL) provide indices of bulk chemical reactivity rather than compound identities. These assays respond to sugars, ascorbate, proteins, and Maillard products as well as to phenolics, preventing attribution of observed changes to individual molecules [29,30]. The two types of indices can also show opposing trends within the same sample. For example, in one kombucha starter, the total phenolic content increased from 372.57 to 416.77 mg GAE/L over 14 d, whereas the total antioxidant capacity decreased from 1.42 to 1.22 mmol Trolox equivalents/L [31]. The reliance on these measurements, together with the inoculum carryover problem discussed in Section 9, largely explains the scarcity of compound-resolved evidence for non-tea kombucha.

Primary metabolite profiles followed a broadly consistent pattern but varied in magnitude and dominant acid. In sweetened black tea, acetic acid increased from 0.65 to 16.57 g/L, and gluconic acid increased from 0.33 to 7.36 g/L over 21 days. Reducing sugars peaked at 8.2 g/L on day 7 before declining to 2.25 g/L, while ethanol peaked at 0.28 g/L on day 7 before decreasing to 0.073 g/L due to oxidation by acetic acid bacteria [29]. Green tea kombucha reached 9.5 g/L acetic acid by day 15, and black tea kombucha reached 2.3 g/L of glucuronic acid by day 12 [3]. Non-tea matrices alter this hierarchy. Kombucha-fermented cow milk predominantly accumulates L-lactic acid at 0.4–0.7 g/100 g, with D-lactate and acetate below 0.06 g/100 g [19]. Common buckwheat kombucha is also lactate-dominant, with L-lactic acid at 16.82 mg/mL, followed by citric (8.65 mg/mL), acetic (7.14 mg/mL), succinic (3.78 mg/mL), oxalic (0.86 mg/mL), and glucuronic (0.86 mg/mL) acids [16]. Soy whey, by contrast, is acetate-dominant, reaching 5.79 g/L on day 6 [32]. Reported glucuronic acid concentrations are particularly difficult to reconcile, ranging from 2.3 g/L in sweetened tea measured by direct HPLC [3] to 132.81 g/L in soy whey measured after pre-column derivatization [32]. A discrepancy of this magnitude cannot be interpreted without harmonized methods and shared reference material. Ethanol is both a metabolic intermediate and a regulated analyte, and its final concentration is determined by strain composition rather than matrix identity. Consortia containing *Zygosaccharomyces bailii* reached 17,000–22,000 ppm, whereas the corresponding consortia containing *Debaryomyces hansenii* remained below 300 ppm [18].

Compounds with the clearest functional rationale can be categorized into several groups. D-Saccharic acid-1,4-lactone, a β-glucuronidase inhibitor frequently invoked to explain the hepatoprotective effects attributed to kombucha, can be quantified using HPLC. Its concentration reached 2.24 g/L in black tea after 21 days [29] and 2.94 g/L in a rationally reconstructed consortium that also increased gluconic acid to 74% of the total acids [17]. Phenolic biotransformation involves changes in composition and abundance. Untargeted UPLC–MS^E^ revealed an increase in phenolics in black tea kombucha (log_2_ fold change, 1.7), driven by the degradation of flavonoids such as nepetin, hesperidin, and catechin 5-*O*-gallate and the accumulation of gallic and cinnamic acids [25]. Buckwheat kombucha, on the other hand, retains a substrate-derived profile dominated by catechin (441.77 mg/L), with protocatechuic, caffeic, and chlorogenic acids and rutin at 35.78–60.44 mg/L [16]. Deglycosylation is the best-documented enzymatic conversion reaction. In soymilk fermented at 28 °C, total isoflavone β-glucosides decreased from 610.65 to 62.60 µg/g dw, whereas aglycones increased from 137.01 to 722.24 µg/g dw. Conversion was faster and more complete at 37 °C, yielding corresponding concentrations of 7.97 and 806.92 µg/g dw, along with complete consumption of raffinose and near-complete consumption of stachyose [15]. Changes in micronutrient and lipid levels are matrix-dependent. Total folate content increased in fermented cow milk (5.41 to 12.58 µg/100 g FW) and almond drink (2.16 to 9.02 µg/100 g FW) but decreased in the coconut drink (4.30 to 2.37 µg/100 g FW). Manganese increased approximately sevenfold in cow milk, and *cis*-9,*trans*-11 CLA increased in fermented regular cow milk [28]. Specialized metabolites may also be generated, retained, or depleted. Broccoli-stalk infusion yielded sulforaphane at up to 31.39 µg/100 mL and sulforaphane-*N*-acetylcysteine at up to 5.37 µg/100 mL, whereas the precursor, glucoraphanin, was no longer detectable [26]. Wine-lees beverages retained 5.60 mg/L total anthocyanins, compared with 1.69 mg/L in grape-pomace beverages [20], and a defined three-strain consortium produced 2.2 mg/L GABA in white tea [33].

Three limitations emerge from this evidence. First, analytical traceability is rarely established in the literature. Reporting units varied across studies (g/L, mg/mL, µg/g dry weight, mg/100 mL), recovery and matrix effects were seldom documented, and none of the reviewed studies used isotope-labeled internal standards or certified reference materials. These limitations undermine the reliability of comparisons of individual compounds across studies. Second, no non-tea study has reported a closed carbon or mass balance linking substrate consumption to the sum of identified products, leaving the proportion of the matrix characterized at the molecular level unknown. Third, undesirable compounds are measured less systematically than those considered beneficial. Aflatoxin M_1_ was detected at 0.17–0.61 µg/L in tea kombucha, with the highest prevalence at the lowest fermentation temperature, whereas aflatoxins B_1_, B_2_, G_1_, G_2_, and ochratoxin A were not detected in 64 samples [34]. Butyric and isobutyric acids have also been detected in adapted whey fermentations [35], but comparable screening has not been applied to most non-tea matrices. Compound-resolved analysis using harmonized methods, together with control design, is therefore a prerequisite for substantiating functional claims about non-tea kombucha products.

## 4. Dairy Fermentation Applications

### 4.1. Fermented Milk and Yogurt-Type Products

Kombucha-derived cultures have been used to produce acidified dairy beverages with yogurt-like sensory and structural attributes (Table 1). These products are more accurately described as fermented milk or yogurt-type products because yogurt is conventionally defined by fermentation with *Streptococcus thermophilus* and *Lactobacillus delbrueckii* subsp. *bulgaricus* [36]. Terminological precision is equally important for the inoculum classification. Most studies added 10–20% (*v*/*v*) cell-containing kombucha broth propagated in sweetened tea, rather than the cellulose pellicle itself, while others used concentrated broth or liquid starters previously propagated in milk. Therefore, product composition reflects both microbial transformation and the transfer of acids, sugars, polyphenols, and solids from the inoculum. Native tea-derived inocula generally acidify cow milk to pH 4.4–4.6 within 6–12 h at 42–43 °C, substantially more slowly than conventional yogurt starters [37,38,39,40]. Increasing the inoculum volume rarely accelerated fermentation and often weakened the texture by diluting milk solids. Starter concentration and prior propagation in milk were associated with faster acidification rates. Vacuum-concentrated liquid starters reduced fermentation time to approximately 3–6.5 h, whereas the use of a milk-based liquid inoculum approximately halved a 12.5-h process [38,41]. However, these studies evaluated fermentation kinetics and product characteristics without strain-resolved community profiling or functional gene expression analysis. Consequently, faster acidification cannot be attributed specifically to ecological selection, reversible physiological acclimation, or heritable genetic adaptation. Temperature remained an independent process determinant; fermentation to pH 4.6 required 13 h 40 min at 37 °C but 8 h 15 min at 42 °C [42]. Thus, prior milk propagation and temperature are empirically supported process variables.

Some kombucha-derived cultures partially metabolize lactose. Enzymatic determinations have reported reductions of approximately 16–30%, with residual lactose of 3.30–4.00 g 100 g^−1^ and accumulation of galactose [19]. L-lactic acid dominated the metabolite profile [19,39], whereas D-lactate, acetate, and ethanol remained low. Therefore, milk fermentation differs markedly from the acetate-rich metabolism associated with tea kombucha, probably because the lactose-rich, strongly buffered dairy matrix favors different metabolic activities within the consortium. Kombucha-derived liquid inocula produced comparable patterns in conventional and lactose-free products, although neither residual lactose nor glucose was depleted sufficiently to justify low-sugar claims without regulatory verification [43].

Acidification generates a coherent casein network. Kombucha-induced gelation began at pH 5.80, earlier than that with the yogurt starter (pH 5.39) or the commercial ABT-7 starter (pH 5.42), and yielded a homogeneous set gel with rheological properties comparable to those of the yogurt-starter product [44,45]. The two reports arose from overlapping experiments and should not be treated as independent replications. After stirring and refrigerated storage, kombucha products showed lower firmness and weaker rheological stability than conventional comparators, demonstrating that set-gel characteristics change during storage [40]. Microbial transglutaminase improves firmness, consistency, water-holding capacity, and yield stress, while reducing syneresis [46,47,48]. These gains resulted primarily from enzymatic protein crosslinking rather than the intrinsic texturizing capacity of the kombucha starter culture.

However, evidence for enhanced nutritional or physiological functionality remains preliminary. The increases in folate and cis-9,trans-11 conjugated linoleic acid reported in fermented cow milk are single-study observations requiring independent confirmation and bioaccessibility assessment [28]. Kombucha-fermented milk also exhibited storage-dependent angiotensin-converting enzyme (ACE) inhibition and assay-dependent antioxidant activity. Disagreement between the DPPH and ABTS results, together with tea-derived reducing compounds, precludes a general claim of improved antioxidant capacity [49]. Stronger molecular evidence was obtained from milk inoculated with kombucha-fermented green tea. Sequencing of isolates recovered from the inoculum identified *Gluconobacter oxydans*-like bacteria and *Brettanomyces anomalus*-like yeasts [50].

Overall, kombucha-derived inocula can partially ferment lactose, produce predominantly L-lactic acid, induce casein gelation, and generate yogurt-like dairy products. Prior milk propagation, starter concentration, and controlled temperature were associated with shorter fermentation times, whereas texture may require protein enrichment or cross-linking. However, the community and molecular mechanisms underlying milk-conditioning effects remain unclear. Moreover, the evidence is concentrated within a small number of related research groups and often lacks composition-matched inoculum controls, strain-resolved community analyses, independent batch replications, and adequately powered consumer studies. Reproducible applications will require a defined starter composition, controls distinguishing fermentation from tea-broth carryover, mass-balanced metabolomics and peptidomics, and validation of safety, shelf life, and physiological effects.

**Table 1:** Representative studies using kombucha-derived consortia in fermented milk and yogurt-type products.

Dairy Matrix	Inoculum Format	Fermentation Process	Principal Findings	Reference
Cow milk containing 1.0 or 2.2% fat	Black-tea kombucha liquid starter; 10–20% (*v*/*v*)	Fermentation at 42 °C to pH 4.4; yogurt-starter comparator	Kombucha fermentation required 130–455 min (approximately 2.2–7.6 h) to reach pH 4.4 and was slower than fermentation with the yogurt starter. Increasing the inoculum above 10% *v*/*v* provided little kinetic benefit; milk fat affected the chemical composition and sensory quality.	[37]
Cow milk containing 0.9–2.2% fat	Kombucha liquid starter, native, vacuum-concentrated, or previously propagated in milk; 10 or 15% (*v*/*v*)	Fermentation at 42–43 °C to pH 4.4–4.5	Concentrated kombucha liquid starters reached pH 4.4 within 3–6.5 h. In a separate milk-adaptation study, traditional tea-derived inocula required approximately 12.5 h to reach pH 4.5, whereas milk-adapted inocula shortened fermentation by approximately two-fold and improved texture and sensory quality, particularly at 10% inoculum.	[38,41]
Cow milk containing 0.9–2.2% fat	Kombucha liquid starter, native, microfiltered, or evaporated; native: 10 or 15% (*v*/*v*); microfiltered: 10 or 15% (*v*/*v*); evaporated: 1.5 or 3.0% (*v*/*v*)	Fermentation at 42 °C to pH 4.5	Lactose decreased by approximately 16–30%, whereas galactose accumulated. L-lactate predominated at 0.4–0.7 g 100 g^−1^, whereas D-lactate, acetate, and ethanol remained low.	[19,39]
Cow milk containing 2.0% fat	Black-tea kombucha liquid starter; 10% (*v*/*v*)	Fermentation at 42 °C; yogurt and commercial ABT-7 starter comparators; measurements during acidification	Kombucha initiates gelation at a comparatively high pH and produces a homogeneous casein network. At pH 4.6, several rheological properties were comparable to those of yogurt.	[45]
Cow milk containing 2.8% fat	Black-tea kombucha liquid starter; 10% (*v*/*v*)	Fermentation at 42 °C to pH 4.5; yogurt and commercial ABT-7 starter comparators; 14-day refrigerated storage	ACE inhibition increased from 46.27% after production to 63.4% after storage. Proteolysis, antioxidant activity, vitamin C content, and sensory properties were starter- and storage-dependent.	[49]
UHT cow milk	Green-tea kombucha liquid starter; 1% (*v*/*v*)	Fermentation at 37 °C for 72 h; LAB comparators; ultrafiltration and LC–MS/MS analysis	The <10-kDa filtrate exhibited 93% ACE inhibition. Twenty-one milk-derived peptides were identified, and three synthetic peptides derived from the kombucha culture fermentation exhibited IC_50_ values of 0.03–0.75 μM.	[50]
Cow milk containing 2.0% fat	Kombucha liquid starter; 10% (*v*/*v*)	Fermentation at 37 or 42 °C to pH 4.6	Fermentation was performed for 13 h 40 min at 37 °C and 8 h 15 min at 42 °C. The lactose conversion kinetics were sigmoidal and strongly temperature-dependent.	[42]
Cow milk containing 2.8% fat	Kombucha liquid starter; 10% (*v*/*v*)	Yogurt and commercial ABT-7 starter comparators; 21-day refrigerated storage	All products were shear-thinning and thixotropic. Kombucha-fermented milk had a comparatively low firmness after gel disruption.	[40]

Abbreviations: ACE, angiotensin-converting enzyme; IC_50_, half-maximal inhibitory concentration; kDa, kilodalton; LAB, lactic acid bacteria; LC–MS/MS, liquid chromatography–tandem mass spectrometry; UHT, ultra-high-temperature.

### 4.2. Fortified and Co-Cultured Fermented Milks

Fortified and co-cultured formulations extend kombucha-fermented milk through two strategies: introducing plant-derived components and combining kombucha with conventional lactic acid starters. The former expands sensory and compositional diversity, while the latter addresses slow acidification. Both complicate causal attribution because cell-containing kombucha liquid also transfers tea-derived acids, sugars, phenolics, and aroma compounds. These studies, together with those on fresh cheese and whey fermentation, are summarized in Table 2.

Most early “fortified” products were indirectly enriched. Kombucha was propagated in sweetened peppermint or stinging nettle infusions, after which 10% (*v*/*v*) of the fermentation liquid was transferred to milk. Peppermint- and nettle-derived inocula acidified milk containing 1.6% fat to pH 4.5 within 9.45–13.5 h; higher temperatures shortened fermentation, while the carrier type altered the water-holding capacity, syneresis, firmness, and viscosity [51]. Kombucha-fermented milk products prepared with stinging nettle and winter savory showed different responses to DPPH radicals and similar hydroxyl radical-scavenging responses [52]. Similarly, the conditions that maximized in vitro responses for wild thyme differed from those that yielded the highest sensory scores [53]. These studies established formulation-dependent differences in acidification, texture, and in vitro antioxidant assay responses. However, because composition-matched cell-free starter controls and milk-plus-unfermented-infusion controls were generally absent, they did not establish whether changes in phenolic content or antioxidant assay values resulted from microbial biotransformation or from compounds introduced with the plant infusion and tea-derived starter liquid.

Fermented dairy beverages were prepared by mixing milk with green tea, sage, or blackberry infusion in a 60:40 (*v*/*v*) ratio and fermenting them with a kombucha culture. The blackberry blend received the highest sensory scores and was the most favored product, whereas the total phenolic content, DPPH radical-scavenging activity, sugars, organic acids, and microbial counts varied during 30 days of storage [54]. As the herbal tea replaced a portion of the milk, the treatments altered the total dry matter, fat, and protein content compared with the control. The high DPPH response may reflect the combined contributions of phytochemicals present in the herbal ingredients, compounds transferred with the inoculum, and fermentation-related changes. A recent comparison of kombucha-fermented milk prepared with sucrose- or honey-grown inocula found that the acceptability of the produced samples was high, although the sensory scores declined as acidity and syneresis increased during storage [55]. The study utilized a 15-member panel and a 9-point hedonic scale to evaluate these sensory properties and determine the consumer acceptability and desirability of the products.

Co-culturing directly targets processing efficiency. A thyme-derived kombucha inoculum combined with ABT-7 acidified milk to pH 4.5 within 3.75–4.5 h at 37–43 °C [56]. Related studies using black tea- or thyme-derived kombucha in combination with ABT-7 reported fermentation within 3.5–4.5 h and interaction-dependent differences in L-lactate and texture during storage [57]. However, neither design included matched kombucha-only and ABT-7-only treatments; therefore, the accelerated acidification cannot demonstrate synergy and may largely reflect the commercial starter. In fermented goat milk, 2% *Lacticaseibacillus casei* combined with 5% kombucha reduced viscosity while maintaining higher sensory acceptance than the 10% kombucha formulation. The 10% kombucha combination increased acetic acid bacterial counts and DPPH activity while producing a thinner, less acceptable beverage due to its watery appearance [58].

Fortification can diversify flavor and composition, whereas co-culture can shorten fermentation to industrially relevant times. However, evidence for microbial synergy and strain-specific host health benefits remains insufficient. Factorial studies should compare each starter alone and in combination, include sterile-inoculum controls, and integrate strain-resolved microbiology with mass-balanced metabolite analysis, storage assessment, and adequately powered consumer testing.

### 4.3. Cheese Production

Evidence for cheese production is confined almost entirely to unripened cow milk cheese developed by one research group. In these studies, the starter was not the cellulose pellicle but 10% (*v*/*v*) cell-containing black tea kombucha broth, generally combined with chymosin and incubated at 35 °C until pH 4.5–4.7. Therefore, kombucha functions as an acidifying microbial inoculum within an acid-rennet process rather than as the sole coagulant.

Initial comparisons established feasibility but produced inconsistent results. Iličić et al. [59] reported that kombucha required 20.25 h to reach the target pH, five hours longer than the commercial XPL-1 starter, and produced a markedly lower cheese yield (10.98 versus 17.66%). The resulting cheese was firmer and more concentrated in terms of solids, fat, and protein. However, its grainy texture and distinctive flavor reduced the sensory score. A later study using a closely related process reported fermentation in 12 h 45 min, 75 min faster than XPL-1, together with acceptable, although still inferior, sensory quality [60]. The reversal in relative acidification performance across studies from the same laboratory indicates substantial sensitivity to inoculum history, milk composition, or process control and precludes a general claim that kombucha accelerates cheese production.

Composition and digestion data also require careful interpretation. Higher protein and fat concentrations in kombucha cheese largely coincided with lower moisture content and yield. Therefore, they did not demonstrate enhanced nutrient retention. During simulated gastrointestinal digestion, kombucha cheese released more soluble protein and reached a slightly higher degree of hydrolysis than its XPL-1 counterpart, and its digest showed greater antioxidant responses in some assays [61]. However, the cheeses differed substantially in their initial composition and were digested at different dilution ratios. Peptides were not identified, and the non-standardized in vitro model did not assess intestinal uptake. Similarly, elevated total phenol and polyphenol values cannot be solely attributed to microbial biotransformation because phenolics were introduced using the tea-based liquid inoculum.

Subsequent studies have shifted from starter feasibility to herb-fortified cheese. Sage and wild thyme dust, essential oils, and extracts increased total phenolic values and assay-dependent antioxidant activity while generally maintaining short-term sensory acceptability [62,63]. In artificially inoculated cheeses, sage preparations enhanced the reduction in *Escherichia coli*, whereas thyme preparations were associated with reductions in *L. monocytogenes* during refrigerated storage [64,65]. These effects cannot be attributed to kombucha alone, as the plant preparations supplied independent antimicrobial compounds. Moreover, ground wild thyme increased the antimicrobial effectiveness against *Staphylococcus aureus*, whereas its dry and supercritical fluid extracts showed no effect. A texture study found that storage time had a stronger influence than the thyme preparation format: firmness declined and the protein network disintegrated over 30 days due to proteolysis [66]. Collectively, the literature demonstrates that kombucha broth can initiate acidification and support the production of an acceptable fresh cheese, but it does not establish a reproducible advantage over defined dairy starters. No comparable studies have validated pellicle inoculation, ripened cheeses, independent starter sources, or industrial-scale manufacture. Therefore, claims of improved nutrition, preservation, or shelf life should remain provisional and clearly separated from the effects caused by tea-broth carryover, moisture concentration, and added herbs.

### 4.4. Whey Fermentation and Valorization

Whey contains lactose, soluble proteins, and minerals, making it a potential substrate for kombucha-derived cultures. Evidence remains limited to several laboratory studies using chemically distinct matrices, including fresh sweet whey, acid or yogurt whey, reconstituted whey powder, and whey protein concentrate solutions. This distinction is important because the fermentation of a formulated powder medium does not necessarily demonstrate the valorization of an industrial whey stream.

Belloso-Morales and Hernández-Sánchez [67] first fermented fresh and reconstituted cheese whey using a black tea pellicle. After 96 h at 32 °C, sweet whey treatments reached approximately pH 3.3 and contained ethanol, lactate, and acetate, whereas residual lactose fell below 12 g/L. Fermentation also shifted the yeast community toward lactose-utilizing species. Nevertheless, ethanol reached approximately 5 g/L, and the beverages were strongly sour, salty, and non-carbonated. Suciati et al. [68] subsequently combined a kombucha cellulose pellicle and cell-containing kombucha liquid inoculum with a reconstituted whey protein concentrate. Lactose decreased only modestly, from 5.27% to 4.36–4.67%, while the fermentation products turned out slightly aqueous and received moderate sensory scores. These studies established feasibility but provided limited evidence of efficient lactose conversion and acceptable product quality.

Recent investigations have indicated that whey fermentation strongly depends on microbial composition and adaptation. Sfoglia et al. [69] inoculated a dilute whey-powder medium with homogenized pellicles, with or without 70 g/L of sucrose. Both treatments were acidified for seven days, and the sucrose-containing treatment reached a higher final pH than the unsupplemented medium. However, when ethanol exceeded 0.5% (*v*/*v*) in both beverages, the microbial composition changed markedly, and lactic acid bacteria were absent from the consortium. The small number of experimental replicates and the absence of sensory assessments further limit product-level interpretation.

Community-level evidence comes from Thibodeau et al. [35], who repeatedly propagated kombucha cultures in sweet and acidic whey. The selected starting culture already contained the lactose-utilizing yeast *Brettanomyces anomalus*, which displaced *B. bruxellensis* and became dominant during the adaptation phase. Adapted cultures acidified Cheddar, Gouda, queso-fresco, and Greek-yogurt whey below pH 4 and consumed, on average, 47.7% of the initial sugars. Therefore, most products were low in lactose rather than being lactose-free. Ethanol generally remained below 0.5% alcohol by volume, although some queso fresco whey batches reached 1.6%, and butyrate formation indicated a potential off-flavor risk. The long fermentation cycle and deliberate selection of a lactose-utilizing consortium also restrict generalization to conventional Kombucha cultures. These findings are consistent with ecological selection or species sorting within the consortium. Concomitant metabarcoding and metabolite profiles showed that community restructuring coincided with fermentation performance; however, they did not establish causal metabolic cross-feeding or altered gene expression.

Other studies primarily focused on beverage formulations. Hatami and Fadaei [70] incorporated 10–20% yogurt whey into a sucrose-sweetened orange beverage; increasing whey and inoculum levels raised microbial counts, but lactose conversion and metabolite production were not measured. The type of sugar altered the acidity, color, soluble solids, and antioxidant properties of a reconstituted whey beverage, although the treatment composition and transferred sugar-derived compounds complicated the attribution to fermentation [71]. Collectively, these studies demonstrate that kombucha-derived cultures can acidify diverse whey matrices, particularly when lactose-utilizing yeasts are present or selected through adaptation processes. The evidence for substantive whey valorization remains incomplete. The literature generally lacks complete carbon balances, organic load reduction measurements, pilot-scale kinetics, shelf-life and safety validation, and techno-economic assessments. Therefore, progress depends on matrix-specific starter selection, reproducible serial-propagation protocols, mechanistic resolution of community restructuring and functional gene expression changes, control of ethanol and off-flavor formation, and direct quantification of lactose, protein, and environmental-load conversion.

**Table 2:** Selected primary studies using viable kombucha-derived inoculum in fortified and co-cultured fermented milks, fresh cheese, and dairy-whey fermentation.

Substrate	Inoculum Format	Fermentation System & Conditions	Key Biochemical Changes	Reported Bioactivity, Technological, and Sensory Outcomes	References
Cow milk containing 1.6% fat	Black-tea- or wild-thyme-derived kombucha liquid starter combined with commercial ABT-7 starter culture (co-culture)	A total of 90 mL of kombucha liquid starter and ABT-7 at 0.0935 g/L were added to 900 mL of milk; fermentation at 37–43 °C to pH 4.5; refrigerated storage for 10 days	Acidification required 3.5–4.5 h. Temperature and tea type jointly influenced L-lactic acid formation, while fermentation temperature had no significant effect on chemical characteristics	Tea type significantly affected the water-holding capacity and textural characteristics, specifically firmness, consistency, cohesiveness, and viscosity index, particularly during storage.	[57]
Cow milk blended 60:40 with green-tea, sage, or blackberry infusion	Preactivated commercial kombucha liquid starter; 10% (*v*/*v*)	Fermentation until pH 4.7/4.6; refrigerated storage for 30 days	Continued post-acidification reduced the pH to 4.1–4.7. The organic acids, sugars, phenolics, and antioxidant responses varied with the herbal matrix, with sage yielding the highest glucuronic acid concentration.	The blackberry formulation exhibited the highest antioxidant activity and received the highest sensory scores, while the unfortified kombucha-fermented milk was the least preferred.	[54]
Pasteurized cow milk (2.8% fat) for unripened fresh cheese	Black-tea kombucha liquid starter; 10% (*v*/*v*)	Chymosin added as the coagulating enzyme; fermentation at 35 °C to pH 4.5; XPL-1 starter used as comparator	Kombucha cheese reached pH 4.5 in 12 h 45 min, compared to 14 h for XPL-1. It had higher dry matter, protein, fat, ash, and total phenolic contents, reflecting differences in metabolic pathways and moisture retention.	Cheese exhibited acceptable values for all examined sensory attributes, although it had reduced sensory quality compared to the commercial XPL-1 standard. Artificially inoculated *Escherichia coli* and *Listeria monocytogenes* declined by 98.31% (1.77 log CFU/g) and 98.98%, respectively, during 30-day storage.	[60]
Fresh sweet, fresh acid, and reconstituted sweet cheese wheys	Black-tea-grown pellicle-associated biomass	Pellicle added at 20 g per 250 mL whey; static fermentation at 32 °C for 96 h	Sweet whey reached a pH of approximately 3.3 and a total acidity of 0.07 mol/L. The residual lactose decreased to below 12 g/L, while lactate, acetate, and approximately 5 g/L ethanol accumulated, and lactose-utilizing microorganisms were enriched.	Fermentation produced strongly sour, salty, non-carbonated beverages with limited sensory appeal.	[67]
Sweet and acid wheys from Cheddar, Gouda, queso-fresco, and Greek-yogurt production	Serially propagated, whey-adapted kombucha liquid starter containing *Brettanomyces anomalus*	Repeated serial propagation in dairy whey; microbial communities and metabolites were characterized by metabarcoding and ^1^H NMR	During serial whey propagation, *B. anomalus* displaced *B. bruxellensis* and became the dominant fungal taxon. Cultures acidified whey to below pH 4 and utilized an average of 47.7% of the available sugars, producing acetate and ethanol.	Serially propagated cultures supported low-lactose, but not lactose-free, whey beverages. Ethanol was generally below 0.5% ABV, although some batches reached 1.6%. Butyrate formation indicates a potential off-flavor risk.	[35]

Abbreviations: ABT-7, commercial starter culture designation; ABV, alcohol by volume; CFU, colony-forming units; ^1^H NMR, proton nuclear magnetic resonance; XPL-1, commercial cheese starter designation.

## 5. Plant-Based Milks and Soy-Derived Foods

### 5.1. Soymilk, Soy Whey, and Tofu

Soybean-derived substrates, including soymilk, soy whey, and tofu, offer a distinctly complex fermentation matrix for the kombucha consortium (Table 3). Unlike tea, which principally supplies sugars and polyphenols, soy introduces glycosylated isoflavones susceptible to β-glucosidase-mediated deconjugation, α-galactosyl oligosaccharides (raffinose, stachyose) amenable to microbial catabolism, lipoxygenase-derived hexanal responsible for beany off-flavors, and heat-labile proteins whose coagulation behavior is pH-sensitive. These features render soy substrates particularly responsive to the diverse enzymatic repertoire of acetic acid bacteria (AAB), lactic acid bacteria (LAB), and yeasts in SCOBY.

Xia et al. [15] reported temperature-dependent fermentation patterns: at 37 °C, lactic acid bacteria predominated and lactic acid accumulated preferentially, whereas at 28 °C, acetic acid bacterial activity was associated with greater acetic acid production. Raffinose was no longer detected after fermentation, and stachyose was substantially reduced under the reported conditions. β-Glucosidase activity was associated with the conversion of glucoside-bound isoflavones to the corresponding aglycones, including daidzein, genistein, and glycitein, as well as increases in the total phenolic content and several vitamins. These compositional changes coincided with higher radical-scavenging and α-glucosidase- and α-amylase-inhibition assay values, particularly at 37 °C. Because digestion, absorption, and glycemic outcomes were not evaluated, these findings support increased aglycone content and in vitro biochemical activity but not improved bioavailability or glycemic benefit.

Peng et al. [22] analyzed the microbial community during kombucha–LAB co-fermentation of soymilk. At a LAB-to-kombucha ratio of 1:1, 32 °C, and 42 h, the dominant bacterial genera were *Lactobacillus* (41.58%) and *Acetobacter* (42.39%), whereas the dominant fungal genera were *Zygosaccharomyces* (38.89%) and *Saccharomyces* (35.86%). The community and metabolite profiles were consistent but did not demonstrate metabolic cross-feeding among the consortium members. Volatile profiling showed increased ester and ketone production, decreased hexanal levels, and attenuation of the beany off-flavor. In a companion study, Peng et al. [72] reported that fructooligosaccharide supplementation supported microbial growth and reduced selected off-flavor compounds. An 84-h fermentation produced the preferred volatile profile; however, supplementation intensified sourness. Therefore, both odor reduction and acidity-related sensory acceptance must be optimized together.

Popielarczyk et al. [73] evaluated kombucha-fermented soymilk at room temperature and reported acidification to pH 4.11. A trained 10-member panel described a more intense taste, aroma, and yogurt-like consistency. These results demonstrate compositional, assay-specific, and sensory changes under the tested conditions; however, they do not establish improved nutritional value or physiological benefits. Tu et al. [32] showed that a kombucha consortium could ferment soy whey into a sensorially acceptable beverage within six days. Importantly, the favorable aroma and sensory profiles reported on day 6 deteriorated with continued fermentation, and over-acidification was evident by day 8. This finding identifies fermentation duration and sensory endpoint as critical control variables for this matrix. The consortium reduced pH, produced organic acids, including acetic and glucuronic acids, altered the volatile profile, and increased chemical radical-scavenging and in vitro antimicrobial assay responses. These findings support laboratory-scale by-product valorization but do not establish its commercial feasibility. As adjacent evidence from biomaterials research, Feng et al. [74] used kombucha-fermented soy whey to produce bacterial cellulose with high crystallinity and water-absorption capacity.

Collectively, these studies show that kombucha-derived consortia can ferment soy substrates, convert isoflavone glucosides to aglycones, reduce raffinose and stachyose, alter volatile profiles, and produce organic acids under the tested conditions. These findings also support further investigation of soy-whey valorization through fermented beverages and tofu coagulant applications. However, these findings primarily represent compositional, in vitro, sensory, and technological outcomes and do not establish improved bioavailability, detoxification effects, physiological efficacy, commercial feasibility, or scale-up readiness. The interpretation of phenolic and antioxidant findings also depends on the inoculum-control design. Where tea-derived liquid starter was used without a composition-matched cell-free starter control, phenolics and other reducing compounds transferred with the inoculum could not be distinguished from compounds released or biotransformed from the soy matrix.

**Table 3:** Reported biochemical, in vitro bioactivity, technological, and sensory outcomes of kombucha fermentation in soymilk, soy whey, and tofu.

Substrate	Inoculum Format	Fermentation System & Conditions	Key Biochemical Changes	Reported Bioactivity, Technological, and Sensory Outcomes	References
Soymilk	Kombucha liquid starter; 5% (*v*/*v*)	Kombucha starter culture; 28 °C and 37 °C	Raffinose was not detected after fermentation; stachyose was substantially reduced; isoflavone aglycone content increased; total phenolics, ferulic acid, chlorogenic acid, and ascorbic acid increased	Higher in vitro α-glucosidase and α-amylase inhibition and higher chemical antioxidant assay values at 37 °C than at 28 °C; sensory quality was also rated higher at 37 °C	[15]
Soy whey (tofu by-product)	Liquid starter:soy whey; 1:10 (*v*/*v*)	Kombucha consortium (*Pichia*, *Brettanomyces*, *Acetobacter*, *Lactobacillus*); 6 days	pH: 4.89 to 3.28 (day 3); acetic acid 5.786 g/L; formic acid 2.496 g/L; total flavonoids: 111.61 ± 2.94 to 268.45 ± 2.20 mg RE/mL (day 6); glycitein ↑ 6.2×, genistein ↑ 3.2× vs. unfermented	Antimicrobial activity against *Staphylococcus aureus*, *Bacillus subtilis*, *Escherichia coli*; DPPH EC_50_ 1.66 mg/mL; FRAP 681.03 µM FeSO_4_; fruity ester aroma (nonanal, undecanal) at day 6; over-acidification by day 8 (score 4)	[32]
Soymilk	Co-culture: liquid kombucha starter + LAB; 4% (*v*/*v*)	LAB:kombucha 1:1; 32 °C, 42 h	Dominant bacteria: *Lactobacillus* 41.58%, *Acetobacter* 42.39%; dominant fungi: *Zygosaccharomyces* 38.89%, *Saccharomyces* 35.86%; LAB 7.48, yeast 6.68, AAB 6.83 log CFU/mL	Hexanol: 30.16% to 8.74% of total volatiles; linalool and 2,5-dimethylbenzaldehyde produced; beany off-flavor substantially reduced	[22]
Soymilk	Liquid starter	Kombucha + FOS (prebiotic); up to 96 h; optimal 84 h	β-Glucosidase activity ↑ 68.10 mU/mL; genistein ↑ 612.41%; DPPH scavenging ↑ 25.02%; hexanal reduced; citric acid and linalool produced	FOS intensified sour taste; promoted yeast and LAB co-growth	[72]
Soymilk	Liquid + pellicle	SCOBY fermentation	DPPH and PCL antioxidant activity ↑ (*p* < 0.05); SFA ↓, MUFA ↑ (*p* < 0.05); most minerals (copper, magnesium, calcium, sodium, and potassium) ↓ (*p* < 0.05); iron, zinc, phosphorus, and manganese ↑ (*p* < 0.05)	Thicker yogurt-like consistency; more intense taste and aroma rated favorably by 10-person trained panel	[73]
Fermented soy whey as tofu coagulant	Liquid, kombucha-fermented soy whey used as a coagulant	Kombucha-FSW; pH 3.78–4.50; 17.5–30% *v*/*v*; 35–85 °C	Optimal FSW parameters: pH 4.02, 22.5% *v*/*v*, 65 °C; SDS-PAGE reported β-conglycinin/glycinin co-aggregation; WHC and yield declined above 22.5% and 65 °C	Tofu with cohesive gel structure, optimal textural and sensory profile at identified optimum	[75]
Tofu soy whey	Liquid starter; 10% (*v*/*v*)	Kombucha consortium; 32 °C, 11 days; 8.5% sucrose, 10% inoculum	Bacterial cellulose (BC) yield: 4.20 g/100 mL DW; crystalline cellulose I structure; high water absorption	Suitable mechanical properties for food packaging and biomedical applications	[74]

Abbreviations: AAB, acetic acid bacteria; BC, bacterial cellulose; CFU, colony-forming units; DPPH, 2,2-diphenyl-1-picrylhydrazyl; DW, dry weight; FeSO_4_, iron(II) sulfate; FOS, fructooligosaccharides; FRAP, ferric-reducing antioxidant power; FSW, fermented soy whey; LAB, lactic acid bacteria; MUFA, monounsaturated fatty acids; SFA, saturated fatty acids. The symbols ↑ and ↓ indicate increases and decreases, respectively.

### 5.2. Other Plant-Based Milks

Beyond soy, kombucha-derived fermentation has been investigated in several nut-derived beverages (Table 4), where substrate composition influences acidification, microbial growth, metabolite formation, and sensory properties. Gülhan [76] fermented an almond-milk formulation with a 10% (*v*/*v*) liquid starter at 25 °C for 14 days. The pH decreased from 6.96 to 3.39, and the total soluble solids declined from 7.0 to 4.7 °Brix. Compared with the green tea kombucha control, the almond formulation showed a higher total phenolic content at day 14. In the almond formulation, acetic acid bacteria and yeast reached their highest counts on day 14, 7.99 and 7.23 log CFU/mL, respectively, whereas lactic acid bacteria reached 7.44 log CFU/mL on day 7. Moreover, Göksu and Bölek [77] incorporated almond whey into black tea kombucha at concentrations of 5–15% (*w*/*v*) and fermented the formulations at 25 °C for 12 days. At 15% incorporation, the final pH was 3.31, compared to 2.85 in the control, and the titratable acidity was 0.60%, compared to 0.77% in the control. This difference may reflect the protein-mediated buffering. Higher microbial counts and antioxidant values have also been reported, but their co-occurrence does not demonstrate that changes in microbial diversity cause the measured antioxidant responses.

Czarnowska-Kujawska et al. [28] evaluated almond- and coconut-based formulations containing added sucrose and green tea infusion. During fermentation to approximately pH 4.6, antioxidant values increased in both formulations, whereas the total PCL response was highest in the coconut formulation. The folate concentration increased in the almond formulation but decreased in the coconut formulation. These findings represent matrix-dependent compositional and assay-specific outcomes, and their nutritional relevance requires evidence from digestion, absorption, or in vivo studies. In a cashew-based formulation, Araujo Filho et al. [78] compared kefir, kombucha, and their combination in a cashew-nut beverage fermented at 28 °C for 72 h. Under the reported conditions, the combined inoculum utilized the measured sugars more extensively than either single-culture treatment, with sucrose, glucose, fructose, and raffinose no longer being detected after 48 h.

Collectively, these studies show that nut-derived matrices can support kombucha fermentation. However, most measurements were obtained during active fermentation or at a single time point. The studies included in this review did not provide a systematic longitudinal assessment of post-fermentation storage stability in nut-based and other plant-based dairy alternatives. Unresolved outcomes include continued post-acidification, ethanol and organic acid accumulation, changes in viable microbial populations and community composition, phase separation, viscosity, rheology, lipid oxidation, volatile profile evolution, and sensory acceptance over time. Future studies should evaluate these outcomes at defined refrigerated storage intervals and under specified packaging and cold-chain conditions before shelf-life or commercial stability claims are made. In plant-based beverage studies, increases in phenolic content and antioxidant assay responses should be interpreted cautiously. Several formulations contained green tea infusion or were inoculated with tea-grown fermentation liquid, whereas composition-matched cell-free starter controls were generally unavailable. Therefore, the measured values represent changes in the complete formulation and do not independently demonstrate that the kombucha microorganisms generated or released the measured phenolic compounds from the plant-based matrix.

## 6. Fruits, Vegetables, Juices, and By-Products

Fruit-based kombucha analogs have been more widely studied than vegetable or by-product matrices, although most remain laboratory-scale sweetened beverages. Juices can support yeast and acetic acid bacteria growth without tea. However, acidity, fermentable sugars, nitrogen, phenolics, and inoculum ecology jointly determine the fermentation process. Therefore, evidence from one fruit–consortium combination cannot be generalized to another.

### 6.1. Fruit and Vegetable Substrates

Red grape juice was inoculated with pellicle and cell-containing broth and acidified from pH 3.95 to 2.90 over 12 days. Phenolic and radical-scavenging measurements peaked after six days, coinciding with the highest sensory acceptance, but declined as over-acidification produced a vinegary beverage [79]. King coconut water required added sucrose and is fermented with a selected acid-producing pellicle inoculum. Fermentation increased several phenolic acids, chemical antioxidant responses, and in vitro α-amylase and α-glucosidase inhibition, although formal consumer testing and physiological validation are lacking [80]. Snake fruit pulp also yielded an acceptable beverage with cultivar-dependent acidity and sensory quality. Approximately 45–47% of the total sugars were consumed [81]. Broader comparisons show that fermentation performance depends jointly on the substrate and the microbial consortium. Eight fruit juices fermented with three kombucha consortia differed in acidification, residual carbohydrate, ethanol, phenolic content, and antioxidant activity [82]. In red araçá, butiá-da-serra, and jaboticaba pulps, ten-day fermentation converted sugars into acetate, ethanol, and glycerol, with ethanol concentrations ranging from 0.1 to 0.7% (*v*/*v*). Shotgun metagenomics associated these changes with communities dominated by *Brettanomyces anomalus*, *Zygosaccharomyces*, *Gluconobacter oxydans*, and *Komagataeibacter* spp. [83]. This integration of community and metabolite data is unusually strong, although culture-specific ecology and variable ethanol production constrain process transferability.

The evidence for vegetable matrices is substantially weaker. A conference study fermented a sucrose-supplemented beetroot suspension before microfiltration; organic acids increased, whereas betacyanin and total polyphenol concentrations decreased markedly during the 12-day fermentation period. However, analysis in duplicate only, inconsistent process reporting, and an emphasis on membrane fractionation make this evidence preliminary [84]. Sauerkraut juice is better characterized but represents a by-product of lactic fermentation rather than an intact vegetable [85]. No controlled study identified in the screened literature used a kombucha consortium to ferment whole or minimally processed vegetables.

### 6.2. Agro-Industrial By-Products

Agro-industrial side streams provide a stringent test of whether kombucha-derived microbial consortia can function as starter inocula beyond tea fermentation. However, the direct fermentation of a by-product must be distinguished from the addition of a residue to conventional tea kombucha. The studies considered here inoculated non-tea media prepared from processing residues with an active SCOBY, mature kombucha broth, or both. Nevertheless, most fermented filtered infusions or clarified liquid fractions were supplemented with sucrose rather than the complete residue. Camara et al. [86] used both SCOBY and mature kombucha liquid to ferment filtered infusions prepared with 10% sucrose and acerola-, guava-, and tamarind-processing residues. All three formulations were sensorially acceptable, with the guava beverage preferred. However, the residues were extracted in hot water with sugar and removed before fermentation, and fruit pulp was introduced during the second fermentation. Therefore, the process valorized an extractable fraction, rather than the original biomass.

Targeted analyses demonstrate that successful fermentation does not necessarily preserve native phytochemicals. A commercial SCOBY fermented broccoli-stalk infusion generated detectable amounts of sulforaphane and sulforaphane-*N*-acetylcysteine, indicating the transformation of glucosinolate-derived compounds [26]. However, the temporal instability of these metabolites and the absence of digestion or absorption endpoints preclude conclusions regarding enhanced bioaccessibility or bioavailability. Similarly, direct fermentation of an orange-peel infusion produced acetic acid with low ethanol concentrations, while the total flavanone content decreased from 11.85 to 5.72 mg/100 mL [87]. The observed reduction in flavanones also indicates that direct infusion fermentation may not ensure the efficient recovery or retention of orange-peel bioactives. In a complementary non-fermentation approach, Chaudhary et al. [88] used ultrasound-assisted NADES extraction to recover constituents from orange peel and *Spirulina platensis* for incorporation into a strawberry–cantaloupe-based clean-label functional beverage. This study highlights the importance of controlled extraction and formulation in converting orange peel residues into functional beverage ingredients. Pasteurized sauerkraut juice also supported direct fermentation with an activated kombucha culture comprising SCOBY and mature broth [85]. The beverage remained sensorially acceptable, although its taste and aroma were rated lower than those of black tea kombucha, and the ethanol content reached 0.86% (*v*/*v*) after nine days. As sauerkraut juice is a product of lactic fermentation, its initial metabolites and biogenic amines must be distinguished from those generated during subsequent SCOBY fermentation.

Winery-side streams constitute the most developed evidence cluster. Grape pomace infusion fermented with a backslopped kombucha consortium showed sugar consumption and production of organic acids and ethanol at different sucrose concentrations, temperatures, and fermentation periods [89]. Reported increases in phenolics, anthocyanins, and chemical bioactivity assay responses warrant cautious interpretation because the effects of extraction, concentration, and fermentation were not completely separated. Balmaseda et al. [90] provided stronger evidence for starter cultures by adapting a homemade SCOBY to a white grape marc infusion before comparing grape marc and tea fermentation. The adapted culture consumed more sugar in the grape marc medium, whereas marc from aromatic Muscat grapes improved the volatile profile. Nevertheless, the relatively low phenolic concentration and the accumulation of acetate and ethanol constrained prolonged fermentation. Using a commercial SCOBY, Canovas et al. [20] fermented grape pomace and wine lees infusions. Wine lees supported greater acetate and ethanol production and retained more anthocyanins than grape pomace. Refrigerated beverages met the microbiological criteria during 21-day storage. However, consumer acceptance was not evaluated, and wine-derived enzymes and other endogenous constituents may have contributed to the substrate-specific response. Although the above studies examined different agro-industrial by-products, they share several authors and related fermentation and analytical frameworks. These studies broadened the range of substrates investigated but did not constitute fully independent validation across research teams.

A related series of studies used a kombucha inoculum to ferment sterilized effluent obtained from grape-must clarification as the sole non-tea medium. They established that temperature, initial sugar concentration, and fermentation duration affect acidification, ethanol, antimicrobial responses, antioxidant measurements, and sensory scores [91,92,93]. These papers provide useful process-level evidence but originate from the same substrate and research group and, therefore, do not represent independent replication. Collectively, these studies demonstrate that viable kombucha-derived consortia can directly ferment aqueous media prepared from diverse agro-industrial side streams. However, complete residue utilization, functional superiority, and industrial feasibility have not yet been established. Because many of these studies fermented only filtered infusions or clarified fractions, the efficient recovery of bioactive compounds from residual plant structures remains an important processing challenge. Xu et al. [94] identified ultrasound-assisted extraction combined with deep eutectic solvents as a promising approach for improving the recovery of bioactive compounds from structurally heterogeneous agro-food wastes, while also emphasizing limitations related to solvent viscosity, downstream purification, solvent recovery, safety, and industrial scale-up. This framework suggests that extraction and SCOBY fermentation could be integrated as sequential valorization operations. However, future studies must distinguish between compounds released during extraction and metabolites subsequently produced or transformed by the microbial consortium. Stronger substantiation will require carbon and solid mass balances, measurement of residual biomass and organic load reduction, defined food-grade inocula, separation of extraction from microbial effects, control of ethanol and over-acidification, and validated sensory, shelf-life, safety, techno-economic, and life-cycle assessments.

## 7. Bakery and Cereal-Based Foods

### 7.1. Bread and Sourdough

The incorporation of kombucha consortia into sourdough fermentation represents a mechanistically distinct approach to bread biopreservation and compositional or technological modifications. Unlike conventional sourdough, which relies on endogenous flour microbiota or defined LAB starters, kombucha-derived systems introduce acetic acid bacteria (AAB), osmotolerant yeasts, and their metabolic by-products, particularly acetic acid, glucuronic acid, and gluconic acid, into cereal matrices (Table 5). Kilmanoglu et al. [31] compared kombucha-based and traditional wheat sourdoughs. Acetic acid accumulation in kombucha sourdough, absent in traditional counterparts, translated into antifungal efficacy, delaying visible mold colonization by one day, with the strongest inhibitory activity against *Aspergillus niger* and *A. flavus*. However, kombucha yeasts adapted poorly to the sourdough environment compared to baker’s yeast, suggesting that biopreservation is primarily attributable to AAB metabolism rather than competitive microbial exclusion. The elevated organic acid content produced a vinegary taste and odor, which reduced sensory acceptance. Lyophilization partially mitigated this limitation, increasing overall acceptability by 9–13% and reducing crumb hardness. Exopolysaccharide concentrations were nonetheless similar across all sourdoughs (1.6–1.7 mg/g), so the improvement cannot be attributed to exopolysaccharide production by the consortium.

Mohd Roby et al. [27] advanced practical applicability through spray-drying encapsulation with gum Arabic, retaining ~90% microbial viability, though the starter still required refreshment before being active for breadmaking. The encapsulated kombucha sourdough starter (EKSS) produced bread with superior loaf volume and markedly lower crumb firmness than baker’s yeast bread. It also delayed *Penicillium* sp. growth to 10 days, compared to 4 days for liquid traditional sourdough bread and 3 days for baker’s yeast bread. Shahrampour et al. [95] evaluated four kombucha variants in whole-wheat lavash bread, identifying green tea kombucha sourdough (GKS) as optimal. GKS achieved the highest phenolic content and most effective phytic acid reduction, likely reflecting enhanced endogenous phytase activity under the lower pH generated by kombucha. GKS bread also received higher sensory scores than traditional sourdough bread for taste, color, and odor. Moreover, Tanveer et al. [96] demonstrated a different strategy using SCOBY derived from black tea kombucha as an inoculant slurry, reducing the starter preparation time from 7–10 days to approximately 16 h. Optimization identified an 80% hydration level as ideal, balancing characteristics such as porosity, starch crystallization, texture, total phenolic content, and viscoelasticity. This study demonstrated that SCOBY biomass can serve as a sourdough inoculant, broadening the conceptual scope of kombucha-based breadmaking.

Collectively, the biopreservative efficacy of kombucha in bread is attributed to acetic acid accumulation, rather than competitive exclusion. The reported compositional changes included higher extractable phenolic values and lower measured phytic acid. The principal limitation remains the sensory penalty associated with elevated acidity and a vinegary aroma. Lyophilization and encapsulation were associated with improved acceptability; however, these approaches only partially addressed the problem and have not been established as general methods for controlling acidity or undesirable odors in bread systems. Future studies should focus on dose–response optimization and quantification of the bioaccessibility of kombucha-derived metabolites through simulated gastrointestinal digestion. Similarly, increases in phenolic content or antioxidant activity in bread prepared with tea-derived kombucha starters should be regarded as fermentation-associated unless a composition-matched cell-free starter control is included, as these may be directly introduced with the inoculum.

### 7.2. Buckwheat Beverages

Buckwheat (*Fagopyrum* spp.) is a gluten-free, rutin-rich substrate, and its fermentation with kombucha consortia generates beverages with compositional profiles distinct from those of tea-based kombucha (Table 5). Al-Wraikat et al. [16] fermented common buckwheat pulp with SCOBY under aerobic conditions, yielding a beverage with a lactate-dominant organic acid hierarchy, a shift from the acetic acid dominance typical of tea kombucha and consistent with substrate-driven selection favoring heterofermentative pathways. The phenolic profile was dominated by catechin and included chlorogenic acid, caffeic acid, and rutin, which are compounds originating from buckwheat rather than tea. Microbial sequencing identified *Komagataeibacter* as the dominant bacterial genus and *Brettanomyces bruxellensis* as the dominant fungal species. Membrane-integrity staining (SYTO 9/PI) indicated damage to all three test organisms, with fluorescence-ratio Pearson coefficients of 96.5% for *Cronobacter sakazakii*, 94.7% for *Escherichia coli* and 81.1% for *Staphylococcus aureus*; these are imaging-assay correlation coefficients rather than log reductions in viable counts. Suffys et al. [23] developed Hakko Sobacha, a roasted buckwheat (kasha) beverage, which underwent aerobic fermentation for 10 days, followed by anaerobic fermentation for 15 days. Substrate concentration (10 versus 50 g/L) profoundly influenced the fermentation kinetics. Higher loading produced greater SCOBY pellicle mass, lower terminal pH, and elevated acetic acid, while keeping ethanol below the EU non-alcoholic threshold. The volatile profile exhibited 54 components during fermentation at 50 g/L, including roasting-derived pyrazines, fermentation esters, and *Brettanomyces*-associated phenols, generating a complex sensory landscape of hazelnut, pineapple, and woody and spicy notes. Consumer evaluation reported a 57% preference for the 50 g/L formulation, and 80% of the surveyed participants indicated their willingness to use it as a soft drink substitute. These responses demonstrate acceptability within the study sample but do not establish commercial viability or functional food status. Therefore, the product should be regarded as a consumer-acceptable beverage prototype requiring storage, safety, scale-up, and market validation.

## 8. Coffee, Cocoa, and Botanical Infusions

### 8.1. Coffee

Coffee infusions contain caffeine, chlorogenic acids, melanoidins, and trigonelline, and added sugars can support SCOBY metabolism. Interest in coffee kombucha has focused on fermentation-induced compositional changes. Watawana et al. [97] reported that brewed coffee fermented with a kombucha consortium (24 ± 3 °C, 7 days) produced a viable acidic beverage reaching pH 4.1 and total phenolic content of 26.4 mg GAE/g. The beverage also exhibited in vitro α-amylase and α-glucosidase inhibition. However, the postprandial glycemic effects were not evaluated. Bueno et al. [98] enriched coffee kombucha with *Lacticaseibacillus rhamnosus* and *L. casei* and reported that both cultures maintained viable counts above 10^7^ CFU/mL after simulated gastrointestinal passage. The added microorganisms were reported at the species level, whereas strain-level safety, in vivo persistence or colonization, and health benefits in the target host were not demonstrated. Accordingly, the product is more accurately described as coffee kombucha supplemented with viable *Lacticaseibacillus* cultures.

Błaszak et al. [99] introduced a bioreactor with a constant air flow rate of 2 L/min during coffee kombucha fermentation, demonstrating that enhanced oxygen availability accelerated overall acidification kinetics, shortened the processing time from 8 to 4 days, and increased SCOBY mass in some variants. However, the oxygen transfer behavior, reproducibility, energy demand, and performance at the pilot scale have not been evaluated. Kim et al. [100] provided a biochemical characterization of green coffee beans fermented with kombucha cultures (0 to 24 h, 30 °C), documenting a decrease in pH, an increase in titratable acidity, and an accumulation of volatile compounds such as acetic acid. Karyantina et al. [101] evaluated the impact of coffee variety and concentration during a 12-day fermentation, showing the highest antioxidant activity at the lowest coffee concentration (2.5 g/250 mL water). In that study, both variety and concentration affected the antioxidant and physicochemical profiles of the final beverage. The substrate base has expanded beyond brewed coffee toward agro-industrial by-product valorization. Sales et al. [102] fermented coffee cascara (dried pulp and skin of the coffee fruit) infusions, using their hydroxycinnamic acid content to produce phenolic-enriched kombucha with distinctive fruity and acidic profiles. Hartini et al. [103] demonstrated that aqueous extracts from spent coffee grounds, the largest mass fraction of coffee processing waste, adequately supported SCOBY growth and produced measurable radical-scavenging activity. These studies support laboratory-scale investigations of coffee byproduct valorization.

### 8.2. Cocoa

Cocoa (*Theobroma cacao* L.) processing generates substantial quantities of underutilized by-products, including pulp mucilage (cocoa honey), bean shells, and placenta, which collectively account for over 60% of the fruit mass. These fractions are rich in fermentable sugars (glucose 4.58% and fructose 3.25% *w*/*v* in pulp), polyphenols, and methylxanthines, rendering them biochemically compatible with SCOBY-mediated fermentation. Recent research has explored cocoa-derived matrices across three substrate categories: pulp exudates, fresh mucilage, and bean shell infusions. Yuliana et al. [104] conducted a detailed sensory characterization using diluted cocoa pulp juice (1:20, 12 °Brix) with 3% (*w*/*v*) SCOBY. They reported a significant effect of fermentation time on sensory attributes by day 6, elevated sourness, a color shift from slightly cloudy white to slightly cloudy brown, and an overall acceptance declined monotonically with fermentation time on a five-point hedonic scale, from 3.92 at day 0 to 2.76 at day 8 (“neither like nor dislike”), with the four-day product (3.84) not differing significantly from the unfermented control.

Moreover, Rodríguez-Castro et al. [105] evaluated fresh mucilage from two Ecuadorian varieties, Nacional Fino de Aroma (NCFA) and CCN-51, fermented on a green tea base with sucrose supplementation at 40–100 g/L. The Nacional variety at 40 g/L sugar supplementation (NCFA40) achieved the highest sensory acceptability (flavor 4.84/5, aroma 4.79/5) with a final pH of 3.87 and 6.12 °Brix, whereas the highest sugar concentrations drove the pH down to 2.20 and resulted in lower palatability scores. These findings show that substrate choice and fermentation parameters directly shape product composition. de Oliveira Duarte et al. [106] fermented cocoa bean shell (CBS) infusions (28 g SCOBY, 10% starter, 25 °C, 13 days) and observed accelerated acidification (pH 3.86–3.16), the highest terminal titratable acidity (0.95 g/100 mL), and LAB counts of 5.42 log CFU/mL, surpassing both tea and blended ferments. Amplicon sequencing showed *Brettanomyces bruxellensis* dominating the yeast community (99.98% in the cocoa bean shell SCOBY and 91.19% in a beverage replicate) and *Komagataeibacter saccharivorans* as the leading bacterium (44.65%), indicating distinct microbial profiles across substrates. These studies demonstrate that cocoa by-products can support laboratory-scale kombucha-like fermentation and yield distinct microbial, and sensory profiles. Higher total phenolic content and antioxidant values represent compositional and in vitro outcomes and do not establish their functional food effect or health benefit.

### 8.3. Botanical Infusions

The SCOBY consortium tolerates a broad spectrum of botanical infusions as sole fermentation substrates, although its performance varies substantially with the phytochemical composition. Comparative trials across six medicinal herbs (peppermint, winter savory, stinging nettle, wild thyme, elderberry, and quince) demonstrated that all supported active fermentation under identical conditions (7% sucrose, 10% starter, and 25 °C). However, fermentation kinetics diverged markedly, with elderberry reaching the target acidity within three days, whereas quince required ten days [107]. Peppermint consistently yielded the highest total phenolic and flavonoid concentrations among the herbal substrates, coupled with sensory acceptance comparable to that of black tea kombucha. Substrate identity governs not only the fermentation rate but also the chemical individuality of the product. Czarnowska-Kujawska et al. [108] showed that SCOBY fermentation of mint, nettle, and blackcurrant leaf infusions increased the total phenolic content by 59%, 85%, and 19%, respectively, with nettle exhibiting an almost ten-fold increase in water-soluble antioxidant capacity. Mineral analysis showed higher measured concentrations of iron, magnesium, and calcium in the fermented beverages, whereas copper decreased, with an approximately two-fold reduction in the nettle beverage.

However, not all botanicals are equally hospitable. White hibiscus calyces imposed a highly acidic environment associated with sluggish fermentation and high residual sugar levels over an 18-day fermentation period [109]. In contrast, *Salvia aegyptiaca* fermented slowly owing to nitrogen deficiency, requiring an extended period of 11 days and yielding a reduced total phenolic content relative to the unfermented infusion [110]. These contrasting outcomes delineate substrate boundaries: adequate nitrogen, moderate baseline acidity, and sufficient fermentable precursors are prerequisites for a productive consortium metabolism. Some botanical substrates produced higher values for selected measured endpoints than black tea comparators under the tested conditions. Rose hip and yellow gentian decoctions supported greater SCOBY biomass accumulation than black tea [111], whereas Olympus Mountain tea kombucha (*Sideritis scardica*) showed higher measured B-vitamin (vitamins B_1_, B_2_, B_6_, B_7_, and B_12_) concentrations and greater in vitro α-glucosidase inhibition [112]. These findings identify a broad design space for the development of fermented beverages but do not establish their functional food status. Across coffee, cocoa, and botanical systems, the measured phenolic concentrations and antioxidant activities may reflect the combined effects of the native substrate, compounds introduced with the starter, extraction during incubation, and microbial metabolism. Without a composition-matched cell-free starter control, the relative contribution of microbial biotransformation cannot be determined.

## 9. Reproducibility and Process Control

**Figure 3:**
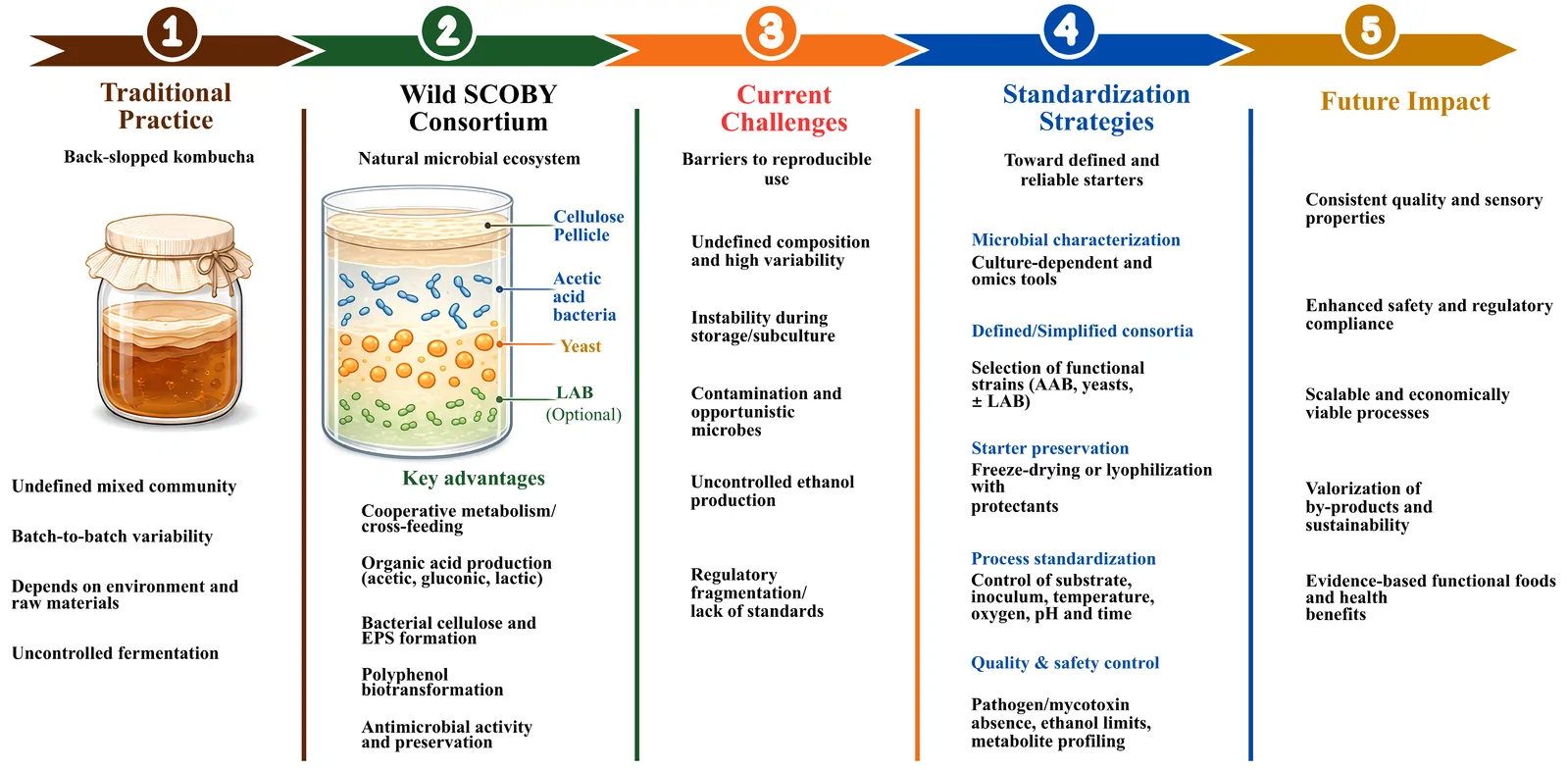
Framework for developing a kombucha SCOBY from an undefined, back-slopped culture into a standardized starter: the wild consortium, barriers to reproducible use, standardization strategies, and target performance attributes.

A central obstacle to deploying kombucha-derived consortia as industrial starters is the instability of undefined, back-slopped SCOBYs, whose composition is not routinely characterized between successive passages. Figure 3 traces the progression from a traditional, undefined culture through the barriers to reproducible use to the standardization strategies developed to address them. Grassi et al. [6] quantified the problem: over 90 days, acetic acid bacteria (AAB) remained stable at 4 °C but became undetectable at room temperature, while *Saccharomyces* species disappeared entirely and *Brettanomyces anomalus* (reported as *Dekkera anomala* in the original study) persisted, converting a multi-genus consortium into a *Brettanomyces*-dominated community. Nevertheless, the total viable yeast counts remained unchanged, masking this compositional collapse. Therefore, bulk enumeration is insufficient to guarantee starter functionality because the metabolic potential depends on the species present rather than the number of surviving cells. In non-tea matrices, where performance cannot be predicted from prior batches, such unmonitored drift renders fermentation irreproducible.

The reporting of inoculum microbiology across the reviewed literature was inconsistent. Many studies provided no community characterization, and those that did used non-equivalent approaches ranging from viable count and isolate-based methods to metabarcoding, shotgun metagenomics, and strain-defined preparations. Because these differ in taxonomic resolution, starter compositions are not directly comparable, and microbial findings should be interpreted at the level of the individual study. Second, the repeatedly reported benefits of substrate adaptation cannot yet be explained mechanistically. The benefit of serial propagation may arise through ecological selection of taxa already present, reversible physiological acclimation, or heritable genomic adaptation, which are not equivalent. Whey propagation provides evidence of community restructuring [35], whereas milk studies report process-level improvements without strain-resolved succession or gene expression data [41,42]. Distinguishing these mechanisms will require absolute enumeration combined with strain-resolved metagenomics, time-resolved metatranscriptomics, and metabolomics across successive passages, together with tests to determine whether the improved phenotype persists after transfer away from the adapting substrate. Another limitation concerns the chemical carryover from tea-derived liquid inocula, which transfers phenolics, organic acids, sugars, pigments, and extracellular metabolites alongside viable cells. When a fermented treatment is compared only with an uninoculated matrix, higher DPPH, ABTS, FRAP, CUPRAC, or PCL values cannot be attributed to microbial release or biotransformation of matrix constituents. Therefore, studies should include three arms: a matrix-only control, a composition-matched control receiving the same volume of cell-free or microbiologically inactivated starter liquid from the same batch, and a viable inoculum. Sterile filtration is preferable to heat inactivation, which may alter phenolic composition and antioxidant activity. The adhering fermentation liquid should be quantified or standardized for pellicle-based inocula. Without such controls, a study can demonstrate fermentation-associated changes but not their microbial origin. The logical remedy is rationally assembled, strain-level-defined starters. Multiple groups have demonstrated that kombucha-like fermentation can be recapitulated using synthetic microbial communities (SMCs) comprising two to six strains. Ferremi Leali et al. [7] selected three strains (*Novacetimonas hansenii*, *Zygosaccharomyces parabailii*, *Brettanomyces bruxellensis*) whose co-culture reproduced the native consortium’s sensory profile while producing the lowest amounts of cellulose matrix. Tran et al. [113] established that the association of *B. bruxellensis* and *Acetobacter indonesiensis* induced kombucha’s characteristic apple-juice aroma. Li et al. [17] identified a two-strain SMC that elevated gluconic acid to 74% of total organic acids by optimizing the yeast-to-bacteria ratio and inoculum size. Chen et al. [18] paired *Komagataeibacter* strains with yeasts, revealing that yeast identity dominated the metabolic trajectory: *Zygosaccharomyces bailii* maximized ethanol at up to 22,000 ppm versus 300 ppm with *Debaryomyces hansenii*. Beyond organic acid programming, Kim et al. [33] developed a defined three-strain starter comprising *Saccharomyces cerevisiae*, *Lactiplantibacillus plantarum, and Acetobacter pasteurianus*. After optimizing the inoculation ratios and initial sucrose concentration, the selected white tea fermentation system produced 2.2 mg/L GABA, providing a laboratory-scale proof of concept for function-directed, defined-consortium fermentation.

Defined consortia may improve control over community composition, storage viability, and batch consistency; however, they do not inherently guarantee safety or reproducibility. Fabricio et al. [114] maintained *Kluyveromyces marxianus* above 6 log CFU/mL through 90 days of refrigerated storage, which demonstrates retained storage viability but not probiotic status, since gastrointestinal persistence and health benefit were not assessed. Liao et al. [115] detected no spoilage organisms in reconstituted cultures, whereas the back-slopped comparator carried several contaminants. Venegas et al. [116] showed that *Saccharomyces eubayanus* genotype reshaped both volatile composition and community structure during co-fermentation. Signorello et al. [117] found that acidification and metabolite production depended on the functional groups included, with the consortium containing yeasts, AAB, and lactic acid bacteria most closely reproducing the original fermentation. Daval et al. [118] further observed that a synthetic consortium fermented reproducibly over several backslopping cycles before changing abruptly, with expansion of *Saccharomyces cerevisiae* and increased ethanol production. Collectively, these remain tea-based laboratory demonstrations, and no defined consortium has been validated in a non-tea food matrix or at the pilot scale.

Standardized deployment also requires preservation in a format that retains viability and fermentative competence. Correia Santana et al. [119] showed that heat dehydration at 42 °C reduced SCOBY bacterial counts by four orders of magnitude and eliminated AAB entirely, whereas freeze-drying preserved AAB at 1.62 × 10^5^ CFU/mL. Phan Van et al. [120] reproduced this hierarchy with a defined preparation (*Komagataeibacter saccharivorans* and *Saccharomyces cerevisiae*): freeze-drying retained 88–90% viability versus 75–79% for spray-drying, and only freeze-dried starters completed acidification (pH 2.65–2.68 versus 4.34–4.79 within 14 d). Viability remained high at 4 °C, whereas 90 days at 30 °C reduced counts by 1.5–1.7 log CFU/g; even so, viability stayed above 7 log CFU/g and fermentation ability was preserved, so refrigeration improves but is not strictly required for retention. Carrier chemistry partially offsets thermal damage: gum Arabic provided spray-dried acetic acid titers (2.67 g/L) comparable to freeze-dried equivalents, against 1.49 g/L for maltodextrin [121]. Freeze-drying with polysaccharide carriers produced the strongest short-term results, although validation across matrices and longer storage periods remains necessary.

Across the reviewed matrices, the reported approaches to limit excessive acidification and undesirable odors fall into five groups. First, fermentation should be terminated at a matrix-specific pH, time, and sensory endpoint rather than run for a fixed duration; dairy fermentations are typically stopped at pH 4.4–4.6 [37,38,40], whereas sensory acceptance peaks at day 6 in red grape juice [79] and in soy whey, which is over-acidified by day 8 [32]. Second, time and temperature should be optimized together because both the acidification rate and the resulting acid profile depend on them [15,42]. Third, the inoculum composition and formulation should be judged based on volatile and acidity endpoints. Lactic acid bacteria co-fermentation reduced beany off-flavor in soymilk [22], fructooligosaccharide supplementation reduced selected off-flavor compounds but intensified sourness [72], and butyrate formation in adapted whey cultures showed that adaptation may itself introduce an off-flavor risk [35]. Fourth, oxygen supply should be coupled to a predefined pH or acidity endpoint rather than being used solely to shorten fermentation [99]. Fifth, starter format and post-fermentation storage require product-specific validation, since lyophilized or encapsulated sourdough starters improved sensory acceptance [27,31], whereas some milk formulations continued to post-acidify during refrigerated storage [54]. Because direct factorial comparisons and pilot-scale validation remain scarce, these measures should be regarded as evidence-based strategies rather than established solutions. Matrix pretreatment is another potentially important process control variable. In their comprehensive review of non-thermal ultrasound applications in functional-beverage processing including fermented beverages such as kombucha, Chaudhary et al. [122] highlighted ultrasound-assisted processing as a strategy for improving substrate extraction and fermentation performance. However, its effects on SCOBY community composition, microbial viability, fermentation kinetics, metabolite production, ethanol formation, sensory quality, and batch reproducibility require systematic evaluation before this approach can be extended to other non-tea matrices. Industrial evaluation requires more than evidence that a kombucha-derived consortium can acidify a specific substrate. Fermentation kinetics, inoculum level and format, temperature, ethanol formation, storage stability, sensory acceptance, microbial identity, experimental scale, and manufacturing validation must be reported together. No non-tea application reviewed here reported this complete set. Fermentation time, temperature, inoculum level, and target pH were the most consistently reported parameters in dairy studies, whereas ethanol monitoring, finished product shelf life, and strain-resolved characterization were intermittent or absent. Sensory assessment ranged from trained-panel and consumer testing to qualitative observations of sourness. All non-tea work remained at the laboratory scale, and consumer acceptance testing did not constitute industrial validation.

## 10. Safety and Regulatory Considerations

The open, non-sterile nature of kombucha fermentation introduces biological, chemical, and mycotoxigenic hazards, the severity of which depends on the process parameters. A Hazard Analysis and Critical Control Points (HACCP)-based assessment cataloged risks spanning bacterial pathogens (*Salmonella* spp., *L. monocytogenes*, *E. coli*, *Clostridium botulinum*), mycotoxin-producing molds (*Aspergillus*, *Penicillium*), and chemical contaminants such as lead leached from ceramic vessels under acidic conditions [123]. The progressive acidification of the fermenting medium, from pH ~5 to ≤2.5 over 7–10 days at 18–26 °C, constitutes a primary intrinsic barrier to pathogen survival. Cullinan et al. [124] quantified this effect using avirulent surrogates in a black- and green-tea blend kombucha fermented at 19–23 °C: mean reductions of 5.86, 5.02, and 4.26 log CFU/mL were achieved for *Salmonella*, *E. coli*, and *Listeria*, respectively, over 14 days, with higher sugar concentrations (80 g/L) attenuating die-off relative to 26–53 g/L. Mycotoxin contamination is a less intuitive, temperature-dependent hazard. Sadok et al. [34] demonstrated that fermentation at 25 °C promoted visible *Aspergillus flavus* growth in ~83% of samples yet yielded only one sample with detectable mycotoxins, whereas 17 °C suppressed mold growth but paradoxically increased mycotoxin prevalence: aflatoxin M_1_ reached 0.51 µg/L, and aflatoxin M_1_ or penicillic acid was detected in 77% of samples. This suggests that cooler fermentations, despite less visible mold, may harbor fewer microorganisms capable of degrading mycotoxins, making macroscopic inspection alone an insufficient safety criterion.

The persistence of active yeasts in unpasteurized kombucha creates a distinct regulatory challenge: post-manufacturing ethanol accumulation. In the United States, beverages exceeding 0.5% ABV fall under the Alcohol and Tobacco Tax and Trade Bureau jurisdiction; however, compliance monitoring is difficult for a living product whose composition evolves on the shelf. Talebi et al. [24] surveyed 18 commercial kombuchas and found that every product exceeded the 0.5% threshold, ranging from 1.12 to 2.00% ABV at the point of purchase. Storage at 22 °C increased concentrations to 1.57% within 14 days; refrigeration (4 °C) permitted a gradual rise over the first 14 days, after which concentrations remained nearly unchanged. An enzymatic assay validated against AOAC SMPR 2016.001 now offers manufacturers a rapid, laboratory-based alternative for monitoring the ethanol content [21]. Globally, regulatory harmonization remains incomplete: Brazil possesses dedicated kombucha legislation (Normative Instruction No. 41/2019) specifying identity and compositional standards; Canada caps ethanol at 1% ABV and mandates stable levels over the shelf life; the Pennsylvania Department of Agriculture imposes a pH range of 2.5–4.2 and prohibits health claims; and the FDA offers no product-specific regulation [125]. This fragmentation has been associated with divergent production practices, such as pasteurization, forced carbonation, or low-ethanol yeast selection, each of which alters the metabolite profile that underpins kombucha’s purported functionality.

Beyond regulatory non-compliance, a small number of clinical case reports have described serious adverse events associated with kombucha consumption. Kovacevic et al. [126] reported hepatitis with markedly elevated transaminase concentrations (ALT, 2163 IU/L; AST, 1288 IU/L) in a patient who had consumed kombucha daily for two years. Sannapaneni et al. [127] documented biopsy-confirmed massive hepatic necrosis in a woman who had consumed both kombucha and wine daily for three months, so attribution to kombucha alone is not possible. Kole et al. [128] described lactic acidosis, hyperthermia, and acute renal failure within 15 h in a patient with newly diagnosed HIV who had consumed 1 L of unpasteurized kombucha. Although *Pichia kudriavzevii* (formerly *Candida krusei*) and *Nakaseomyces glabratus* (formerly *Candida glabrata*) were isolated from the beverage, the patient showed no evidence of a fungal infection. Together with a published review of the reported adverse effects [129], these reports identify potential hazards but do not establish incidence or causality. Therefore, particular caution may be warranted with high or chronic intake and in immunocompromised individuals, although the available evidence is insufficient to define specific clinical risk groups or support a general recommendation to avoid kombucha.

Human evidence on health effects should be interpreted chronologically. The search by Kapp and Sumner [130] was conducted in July 2018 and identified one uncontrolled human study and no controlled clinical trials. Fraiz et al. [131] subsequently included eight human intervention studies published between 2022 and 2024, comprising two non-randomized pre–post studies and six randomized controlled trials; blinding was frequently limited, and the findings were heterogeneous with respect to study populations, kombucha formulations, comparators, intervention duration, and measured outcomes. Therefore, the two reviews are complementary rather than contradictory. Nonetheless, the current human evidence remains insufficient for firm conclusions, and none of the included trials examined non-tea applications.

## 11. Conclusions and Future Directions

The reviewed evidence shows that viable kombucha-derived consortia can act as fermenting agents in food matrices other than sweetened teas. In dairy, soy, other plant-based, fruit and by-product, cereal, coffee, cocoa, and botanical systems, these consortia lower pH within the periods reported for each matrix, produce characteristic organic acid profiles, and, where measured, alter volatile composition. Several systems also exhibit specific technological functions, such as casein gelation in milk, conversion of isoflavone glucosides to aglycones in soy, coagulation of tofu using fermented soy whey, and suppression of mold growth in bread through acetic acid accumulation. These findings support the use of this consortium as a multifunctional starter candidate.

**Figure 4:**
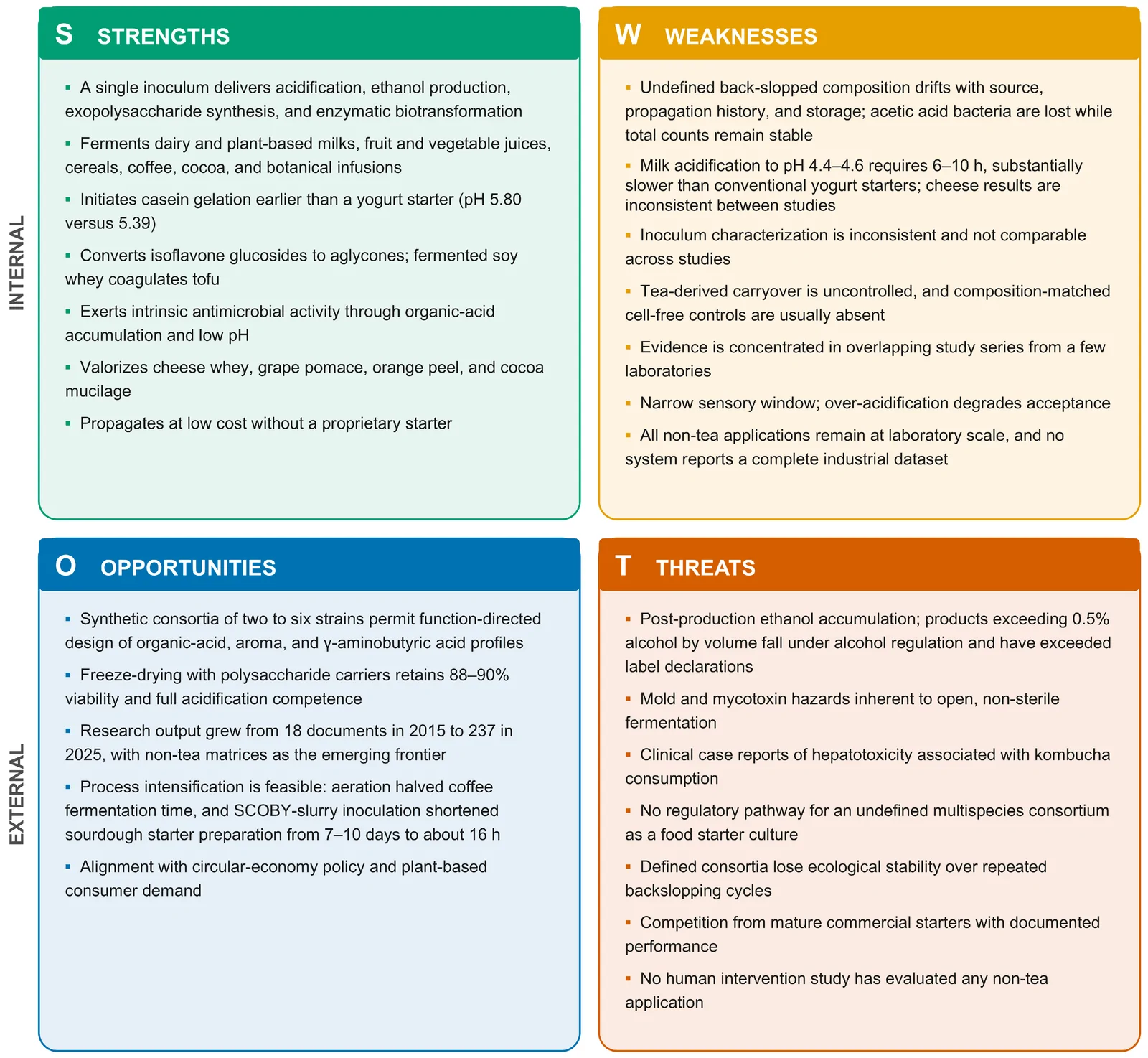
SWOT analysis of the kombucha SCOBY as a starter culture for non-tea food matrices. Strengths and weaknesses (**upper row**) are the internal properties of the microbial consortium, and opportunities and threats (**lower row**) are the external technological, regulatory, and market conditions governing its industrial translation.

The assessment above is summarized as a SWOT analysis in Figure 4, separating the intrinsic properties of the consortium from the external conditions affecting its industrial use. The demonstrated strengths are technological, and the weaknesses identified here follow from a single limitation: no defined and preserved starter has been validated in any non-tea matrix.

The same evidence does not support technological readiness. The reported outcomes were obtained at the laboratory scale, in most cases with an undefined culture whose composition was not characterized, and frequently without controls capable of separating microbial activity from the material introduced with the inoculum. Compositional data and in vitro assay values have often been interpreted as nutritional or physiological benefits, which they do not demonstrate. Several application areas also depend on connected series of studies from a small number of laboratories; therefore, the number of publications exceeds the number of independent validations.

Therefore, further studies are needed to consolidate the existing findings before the range of substrates is extended. Starter cultures should be defined at the strain level and supplied in a preserved format, for which fermentative competence, and not viability alone, is verified after storage. Each target matrix requires separate validation of fermentation kinetics, endpoint control, ethanol formation, safety, shelf life, and sensory acceptance because the performance established in one substrate does not transfer reliably to another. Studies should apply the control design and report the inoculum format and characterization method as standard, so that the results become comparable between laboratories. Analytical practice requires the same consolidation: compound-resolved quantification with internal standards and reference materials, consistent units, and closed carbon or mass balances are prerequisites before compositional changes can be compared between laboratories or attributed to the consortium. Claims should correspond to the evidence obtained, as compositional change, in vitro activity, bioaccessibility, bioavailability, physiological efficacy, and clinical benefit are distinct categories, and only the first two are currently supported for non-tea kombucha products. Until defined starters are validated in the target matrices, preferably at the pilot scale and by independent research groups, the kombucha SCOBY should be regarded as a technologically versatile candidate starter whose functional benefits remain unsubstantiated in humans.

## Figures and Tables

**Table 4:** Kombucha SCOBY fermentation of different plant-based milks.

Substrate	Inoculum Format	Fermentation System & Conditions	Key Biochemical Changes	Reported Bioactivity, Technological, and Sensory Outcomes	References
Almond milk (with green tea)	Liquid starter; 10% (*v*/*v*)	25 °C; 14 days	pH 6.96 to 3.39; acetic acid 21.22 g/L; glucuronic acid 2.84 g/L; lactic acid 2.30 g/L; gluconic acid 3.70 g/L (peak day 7); citric acid 0.83 g/L; ethanol 0.62–0.74 g/L; TSS 7.0 to 4.7 °Brix; TA increased 3–3.5× vs. control	TPC 611.66 mg GAE/L; DPPH 96.90% (5× vs. green tea kombucha); CUPRAC 1.28 mmol Trolox/g (2.5×); AAB 7.99, yeast 7.23, day 7 LAB 7.44 log CFU/mL; sensory acceptability evaluated on a 9-point scale	[76]
Almond whey in black tea (5–15% *w*/*v*)	NR, reported only as “SCOBY”	SCOBY; 25 °C; 12 days	pH 2.85–3.31 (concentration-dependent); enhanced LAB, AAB, and yeast counts vs. control	TPC 426.22 mg GAE/L (15% whey, day 12); DPPH 83.43%; ABTS 83.70 µM Trolox/mL; sensory acceptability > 3/5	[77]
Almond and coconut drinks	Liquid + pellicle	SCOBY; room temperature; ~56 h (to pH 4.6 endpoint)	Folate ↑ 4× in almond (2.16–9.02 µg/100 g); folate ↓ in coconut; Mn content changed significantly; pH-driven acid accumulation	DPPH ↑ 3× (plant drinks); total PCL highest in coconut kombucha; sensory acceptability score was −0.6 for coconut	[28]
Cashew nut beverage	Liquid starter	5 mL kombucha inoculum per 100 mL beverage; 28 °C; 72 h	pH 4.75; glucuronic acid concentration decreased to 2.9788 mg/mL; acetic acid ~3.0 mg/mL	Anxiolytic-like response reported in a zebrafish model; no acute toxicity observed under the tested conditions	[78]

Abbreviations: AAB, acetic acid bacteria; ABTS, 2,2′-azinobis(3-ethylbenzothiazoline-6-sulfonic acid); CFU, colony-forming units; CUPRAC, cupric ion-reducing antioxidant capacity; DPPH, 2,2-diphenyl-1-picrylhydrazyl; GAE, gallic acid equivalents; LAB, lactic acid bacteria; TA, titratable acidity; TPC, total phenolic content; TSS, total soluble solids. The symbols ↑ and ↓ indicate increases and decreases, respectively.

**Table 5:** Key outcomes of kombucha and SCOBY fermentation in bakery and cereal-based food matrices.

Matrix	Inoculum Format	Kombucha System	Fermentation Conditions & pH	Key Metabolites & Bioactive Compounds	Reported Bioactivity, Technological, and Sensory Outcomes	References
Wheat sourdough bread	Liquid starter (black-tea kombucha beverage)	Black tea kombucha (14 d, 28–30 °C) as sourdough starter	SD: 24 h, 30 °C; Bread: 200 °C, 25 min; pH 2.83 (kombucha beverage), initial kombucha SD lower than TS, final SD pH 4.20–4.31.	Acetic acid 9.44 g/L; Glucuronic acid 2.06 g/L (day 7 peak); TPC 416.77 mg GAE/L	Mold delayed to day 5 vs. day 4 (control); inhibition of *A. niger*, *A. flavus*; lyophilization improved acceptability 9–13%; vinegary aroma noted	[31]
White wheat sourdough bread	Encapsulated dried starter (EKSS)	Encapsulated kombucha sourdough starter (EKSS); spray-dried with gum Arabic; 90.27% LAB, 89.52% yeast survival	Dough Leavening: 8 h, room temperature (28 ± 2 °C); Baking: 180 °C, 20 min; pH 5.10 (EKSS bread), 6.06 (BY control bread)	Acetic acid 0.18 mmol/L; Gluconic acid 0.10 mmol/L; Ethanol (0.14 mmol/L); Trehalose; Riboflavin, Pyridoxine, Tryptophan, Alanine, Anserine, and α-aminobutyric acid (0.04 mmol/L) unique to the kombucha starter (LKSS)	Loaf volume 976.7 mL; specific vol. 4.38 mL/g; firmness 116.07 g vs. 316.67 g (LTSS); *Penicillium* sp. growth was delayed to day 10; shelf-life + 2 day; highest taste & overall acceptability (*p* < 0.05)	[27]
Whole-wheat lavash bread	Refreshed kombucha sourdough starter (liquid-derived); 20% (*w*/*w*)	Green tea kombucha sourdough (GKS); 3-stage refreshment to robust dominant flora	SD: 24 h × 3 stages, 30 °C; Bread: 180 °C, 15–20 s; pH 3.8 (SD), 5.46 (dough)	TPC 164.36 mg GAE/100 g (SD), 21.22 mg gallic acid/100 g (bread); DPPH: 86.94% (SD), 58.61% (bread); Phytic acid 32.46 mg/100 g (compared with 39.05 control); TA (SD): 0.77 g/100 mL	TVC (Day 3): 4.59 log CFU/g (vs 6.28 control); Shelf-life: Extended by 1 day (5 day vs. 4-day control); Antifungal: High inhibition compared with *Aspergillus niger* and *Aspergillus flavus*; highest sensory scores for taste, colour, odour, and overall acceptance	[95]
Whole-wheat sourdough bread	9.1% (*w*/*w*) Pellicle-associated biomass + 23% (*w*/*w*) Mature starter per dough	Ground SCOBY (*Komagataeibacter xylinus* + *Brettanomyces bruxellensis*) slurry as inoculant	Starter: 16 h at 32 °C; Dough: 120 min fermentation; Oven: 200 °C for 5 min, then 200 °C for 55 min; pH: SCOBY was pH 2.5 (Bread pH NR); Hydration: 70–90% tested	TPC was 1.34 mg/g for 70% hydration (SB70) and reached 1.54 mg/g for 75% hydration (SB75); higher hydrations (80–90%) had similar, elevated TPC values; moisture content may enhance phenolic solubility and extractability	Optimal at 80% hydration; fermentation time reduced from 7–10 d to 16 h; FTIR reported hydration affected gluten structure and starch gelatinization; SB80 showed the highest initial resistance to deformation and enhanced apparent porosity/texture; sensory analysis NR	[96]
Common buckwheat beverage	Liquid + pellicle/biofilm; 3% (*w*/*v*)	SCOBY (*Komagataeibacter* dominant; *D. bruxellensis*, prevailing among fungi)	Aerobic, day 10, 25 °C	Citric acid 16.82 mg/mL; L-lactic acid 8.65 mg/mL; Acetic acid 7.14 mg/mL; Catechin 441.77 mg/100 mL; Caffeic acid 60.44 mg/100 mL; Chlorogenic acid 56.56 mg/100 mL; Rutin 38.61 mg/100 mL	Antibacterial: *C. sakazakii* 96.5%, *E. coli* 94.7%, *S. aureus* 81.1% reduction; histological findings consistent with gastrointestinal and hepatic protection in a murine model; taste profile altered by grain fermentation	[16]
Roasted buckwheat beverage (Hakko Sobacha; 10 and 50 g/L kasha)	10% (*w*/*w*) Liquid starter + 1% (*w*/*w*) SCOBY pellicle	Commercial SCOBY (Fairment, Germany); two-stage fermentation (aerobic F1 + anaerobic F2)	F1: 10 days, 25 °C (aerobic); F2: 15 day, 25 °C (anaerobic); pH 5.76–3.18 (50 g/L)	Acetic acid 7.13 g/L; Lactic acid 0.33 g/L; Ethanol 0.89% *v*/*v* (50 g/L, day 25); 54 VOCs; final 4-ethylguaiacol 116.18 ppm; final esters 335.34 ppm	SCOBY pellicle 12.47 g wet (50 g/L); ethanol below EU non-alcoholic limit; 57% consumer preference for 50 g/L; 80% willing to substitute daily for soft drinks; hazelnut–pineapple–spicy aroma	[23]

Abbreviations: BY, baker’s yeast; CFU, colony-forming units; DPPH, 2,2-diphenyl-1-picrylhydrazyl; EKSS, encapsulated kombucha sourdough starter; EU, European Union; F1, first-stage fermentation; F2, second-stage fermentation; FTIR, Fourier-transform infrared spectroscopy; GAE, gallic acid equivalents; GKS, green-tea kombucha sourdough; LAB, lactic acid bacteria; LKSS, liquid kombucha sourdough starter; LTSS, liquid traditional sourdough starter; NR, not reported; SB70–SB90, SCOBY breads prepared at 70–90% hydration; TA, titratable acidity; TPC, total phenolic content; VOCs, volatile organic compounds.

## Data Availability

No new data were created or analyzed in this study. Data sharing is not applicable to this study.
